# Multifunctional Membranes for Simultaneous Oil/Water Separation and Organic Pollutant Removal: A Review

**DOI:** 10.3390/membranes16080278

**Published:** 2026-08-19

**Authors:** Zengqing Kang, Yutong Zheng, Tao Wang, Huan Chen, Hua Dong, Junda Liu

**Affiliations:** College of Materials and Chemistry & Chemical Engineering, Chengdu University of Technology, Chengdu 610059, China

**Keywords:** oil/water separation, catalytic degradation, multifunctional membranes, synergistic mechanisms, membrane fouling mitigation

## Abstract

Oily wastewater commonly contains dissolved organic contaminants such as dyes, antibiotics, and phenolic compounds. Conventional stepwise treatment processes involve complex operation, high energy consumption, and severe membrane fouling. Multifunctional membranes integrating oil/water separation, pollutant adsorption or catalytic degradation, and membrane self-cleaning provide a promising solution for treating complex oily wastewater. This review summarizes recent advances in multifunctional membranes based on metal oxides, two-dimensional (2D) materials, three-dimensional (3D) porous structures, and biomass-derived materials. Key strategies, including micro and nanoscale structure regulation, wettability control, interlayer channel optimization, heterojunction construction, and active site engineering, are discussed together with the synergistic mechanisms involving oil/water separation, adsorption enrichment, photocatalysis, and Fenton reactions. Approaches for improving membrane flux, separation efficiency, degradation activity, antifouling performance, and cycling stability are also reviewed. Finally, challenges related to scalable fabrication, adaptability to real wastewater, long-term stability, and standardized evaluation are outlined, providing guidance for the design and practical application of multifunctional membranes.

## 1. Introduction

### 1.1. Characteristics and Treatment Challenges of Oil-Containing Composite Polluted Wastewater

With the continuous advancement of industrialization, the volume of oil-containing wastewater discharged by industries such as petrochemicals, textile dyeing, pharmaceutical production, machinery processing, and catering services has been continuously increasing. Oil-containing discharges from industrial activities and offshore spill events have therefore made oily wastewater a persistent target for global water remediation [1,2,3]. The oil phase in this type of wastewater exists in various forms such as floating oil, dispersed oil, emulsified oil, and dissolved oil. These streams can disrupt aquatic gas exchange and ecological balance and can transport co-contaminants through food webs, motivating sustained attention to their environmental and health implications [4,5].

It is worth noting that industrial wastewater in reality rarely exists in the form of a single oil phase pollution. It often forms a complex composite pollution system with various soluble organic pollutants. Among them, dye pollutants are widely present in textile and dyeing industry wastewater, characterized by high color intensity, difficulty in degradation, and strong toxicity; antibiotic pollutants mainly originate from pharmaceutical and livestock breeding wastewater, and are prone to induce drug resistance in environmental microorganisms; phenolic pollutants mainly come from petrochemical and coal chemical industries, and have strong toxicity and difficulty in degradation [6,7,8]. The composite pollution system comprising oil phases and soluble organic substances exhibits additive toxic effects, while the oil phase can encapsulate or adsorb organic pollutants, thereby shielding them from contact with treatment agents. The conventional stepwise treatment paradigm, “oil separation first, followed by organic degradation”, involves lengthy process chains, numerous equipment units, large footprints, and high energy consumption as well as operation and maintenance costs. Moreover, poor integration between unit operations and mismatched treatment efficiencies further undermine overall performance, rendering this approach inadequate for meeting the contemporary demands of intensive, efficient, and low-cost wastewater treatment [9,10]. These limitations have motivated integrated membrane designs that couple phase separation with adsorption or catalytic degradation rather than relying on a single treatment function [11,12,13].

### 1.2. Application Bottlenecks of Traditional Membrane Separation Technology

Membrane separation technology has been rapidly developed and widely applied in the field of oil-containing wastewater treatment due to its significant advantages such as high separation efficiency, simple operation, and no secondary pollution. According to the separation principle and membrane pore size, microfiltration, ultrafiltration, nanofiltration, and other pressure-driven membranes, as well as superwetting separation membranes, can achieve efficient separation of oil and water phases through size sieving and wetting selectivity, especially for the removal of emulsified oil, effectively making up for the shortcomings of traditional physical separation technologies [1,14,15].

However, traditional separation membrane technology still faces two core bottlenecks in practical applications. The first is the serious membrane fouling problem: oil droplets in oil-containing wastewater are prone to adsorb and adhere to the membrane surface and form an oil layer, and even enter the membrane pores to cause blockage, leading to continuous decline in membrane flux and continuous decrease in separation efficiency. To restore membrane performance, frequent physical or chemical cleaning is required, which not only increases operation and maintenance costs and operational complexity, but also causes membrane material aging and damage, significantly shortening the membrane’s service life [5,16,17]. The second is the limitation of single functionality: traditional separation membranes can only achieve physical separation of oil and water phases, but cannot remove soluble organic pollutants such as dyes, antibiotics, and phenols in the water. The content of soluble pollutants in the membrane effluent often fails to meet the discharge standards, and subsequent deep treatment units must be added, which undoubtedly increases the complexity and overall investment and operation costs of the entire treatment process [7,12,18].

To solve the problem of organic pollutant removal, some studies have attempted to combine catalytic materials with membrane separation technology [2,13,19,20,21,22,23], but early coupling systems mostly adopted a separate design of “membrane separation unit + independent catalytic unit”, which essentially did not break away from the framework of step-by-step treatment, and had problems such as long process, large mass transfer resistance, and limited catalytic efficiency. In addition, the integrated membranes that directly load catalytic materials on the surface of separation membranes also often face technical bottlenecks such as insufficient exposure of catalytic active sites, difficulty in synergistic matching of separation and catalytic functions, and limited effectiveness in alleviating membrane fouling, making it impossible to truly achieve “one-step” efficient treatment of oil-containing organic composite wastewater [9,24,25].

### 1.3. The Proposal and Core Value of Multifunctional Separation Membranes

In response to the technical pain points of traditional separation membranes, such as single functionality and easy pollution, a new type of multifunctional composite membrane that integrates efficient oil/water separation and catalytic degradation of organic pollutants has emerged, becoming a research hotspot in the current water treatment membrane field [26,27,28]. This type of membrane material constructs a special wettability functional layer on a porous support substrate while simultaneously loading catalytic active components, integrating separation and catalytic functions within the same membrane unit, achieving an integrated treatment mode of “one-time filtration, simultaneous treatment”, and demonstrating outstanding technical advantages and application value.

Specifically, the core advantages of multifunctional separation membranes are reflected in three aspects. First is the functional integration advantage: completing the efficient separation of oil phases and the catalytic degradation of soluble organic pollutants within a single membrane module, without the need to add additional deep treatment units, which can greatly simplify the treatment process, reduce equipment land occupation, lower investment and operating costs, and meet the needs of intensive wastewater treatment [29,30,31]. Second is the synergistic enhancement advantage: the catalytic degradation process can not only remove soluble organic pollutants in the water body but also decompose organic pollutants adhering to the membrane surface and pores, giving the membrane material self-cleaning ability, effectively alleviating membrane fouling, improving the operational stability and service life of the membrane, and reducing the frequency of cleaning and operation and maintenance costs [24,25,32]. Third is the scenario adaptability advantage: according to different catalytic mechanisms, multifunctional membranes can be divided into various technical systems such as photocatalytic type, Fenton-like catalytic type, and adsorption–catalysis synergistic type, which can be flexibly selected according to the water quality characteristics, treatment requirements, and on-site conditions of the wastewater, adapting to the wastewater treatment needs of different industries and different scenarios [22,23,33].

From the perspective of mechanism, the operation process of multifunctional separation membranes includes two core zvenos: separation and catalysis. In the separation aspect, by constructing micro/nano hierarchical structures and hydrophilic/hydrophobic functional groups on the membrane surface, special wettability such as superhydrophilicity/underwater superoleophobicity or superhydrophobicity/superoleophilicity can be achieved. Combined with the size sieving effect of membrane pores, this enables efficient separation of different forms of oil phases [2,34,35]. In the catalytic aspect, the loaded catalytic active components can generate reactive oxygen species such as hydroxyl radicals, sulfate radicals, and superoxide radicals under external stimuli such as light and oxidants. These species can gradually mineralize soluble organic pollutants in water into carbon dioxide, water, and small molecular inorganic compounds through oxidation reactions, achieving harmless treatment of pollutants. At the same time, the catalytic reactions on the membrane surface can also decompose adhered organic pollutants, restoring the membrane’s separation performance and achieving self-cleaning effects [22,36,37].

The development goal of multifunctional separation membranes is to achieve the unification of high flux, high separation efficiency, high degradation activity, strong antifouling properties, and long-term cycling stability through material structure design, interface regulation, and functional integration. This aims to solve the problems of complex processes, low efficiency, and high costs in traditional treatment technologies, providing a new technical pathway for the efficient, low-cost, and intensive deep treatment of complex oily organic wastewater [10,25,26,28,38].

In analyzing Table 1, it is apparent that the performance gap between conventional and multifunctional membranes is not primarily one of separation efficiency: a commercial Al_2_O_3_ microfiltration membrane already reaches 98.5% rejection [39], comparable to the >99% reported for TiO_2_-based multifunctional membranes. The decisive differences lie in two other columns, removal of dissolved pollutants and antifouling behavior, which together define the functional rather than the separative advantage of multifunctional membranes.

The comparison highlights a fundamental trade-off between technological maturity and functional integration. Conventional membranes are industrially mature but are essentially limited to physical separation, whereas multifunctional membranes integrate separation, pollutant degradation, and self-cleaning at the expense of remaining at the laboratory stage. This suggests that the main obstacle to practical application lies less in separation performance than in long-term stability, scale-up, and life-cycle assessment.

### 1.4. Overview of Research Progress and Framework of This Review

In recent years, researchers have conducted extensive systematic studies on the material construction, performance optimization, and mechanism analysis of multifunctional separation membranes. Recent studies now span antifouling and antibacterial membranes as well as MXene-polymer remediation platforms, demonstrating the expanding academic and application scope of this field [38,47,48]. In terms of material systems, from the earliest widely studied metal oxide-based photocatalytic membranes such as TiO_2_ and ZnO, to two-dimensional (2D) nanomaterial-based separation membranes such as graphene oxide, MXene, g-C_3_N_4_, and metal–organic framework (MOF), and further to three-dimensional (3D) porous frameworks, biomass-based, and other new functional membranes, the material systems have continuously expanded, and performance has continuously improved [14,21,22,49]. In terms of mechanism research, researchers have deepened their understanding of core scientific issues such as the interfacial interaction principles of oil/water separation, the charge transfer mechanisms of catalytic degradation, and the synergistic effects of adsorption–catalysis, providing theoretical support for the rational design of materials [7,22,27]. In terms of performance optimization, through strategies such as nanostructure regulation, heterostructure construction, surface modification, and interface engineering, the separation flux, degradation efficiency, antifouling properties, and cycling stability of membrane materials have been significantly improved. Recent work also combines antifouling and antibacterial surface engineering [10,38] with ultraviolet (UV)-responsive wettability control [50], further expanding the application boundaries of multifunctional membranes.

Although significant progress has been made in related research, there is still a lack of systematic sorting and summarization of the design strategies, mechanisms, performance systems, and application prospects of multifunctional separation membranes. Based on this, this review, with the core theme of multifunctional oil/water separation membranes, classifies and elaborates on the design ideas and research progress of different systems of multifunctional membranes including metal oxide-based, 2D material-based, 3D structure-based, and biomass-based membranes from the perspective of material construction. It also deeply analyzes the coupling mechanisms of oil/water separation, catalytic degradation, and adsorption–catalysis synergy. It systematically summarizes the performance optimization paths for high flux, antifouling enhancement, and stability improvement. Furthermore, it analyzes the practical challenges and future development directions of the field in combination with the current research status. This paper aims to provide a systematic theoretical reference and technical guidance for the development and industrial application of new, efficient multifunctional separation membranes, promoting the continuous evolution of water treatment membrane technology toward integration, intelligence, and environmental friendliness.

**Figure 1:**
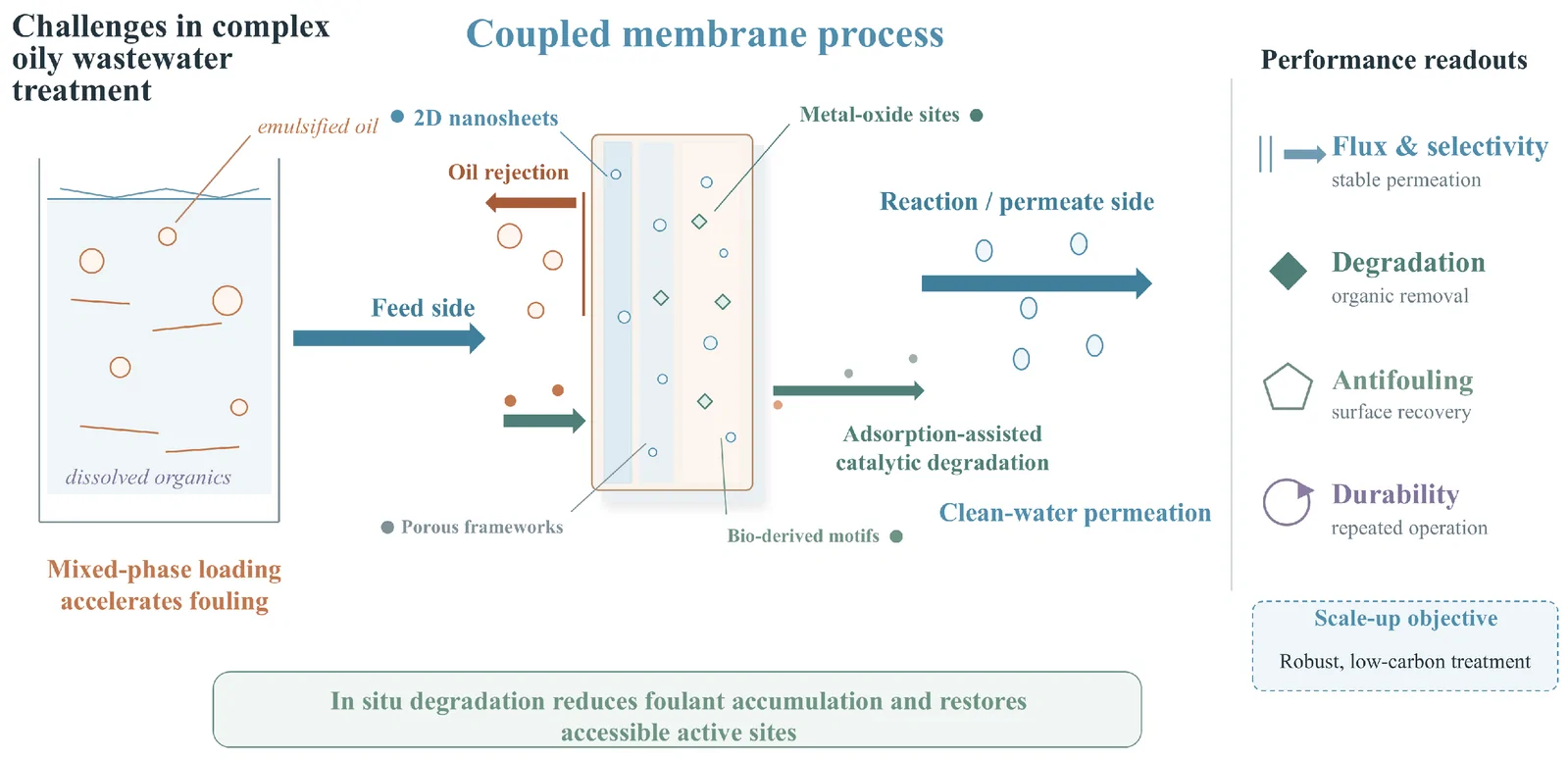
Conceptual framework of multifunctional membranes coupling oil/water separation with catalytic degradation. The scheme links mixed-phase pollutant loading to membrane-mediated oil rejection, interfacial catalytic degradation, clean-water permeation, antifouling performance, durability, and scale-up requirements.

The conceptual framework in Figure 1 organizes this review: the material-construction layer corresponds to Section 2.1, the interfacial and structural regulation layer to Section 2.2, the coupled separation–catalysis step to Section 2.3, and the outer ring of antifouling, durability and scale-up to Section 2.4 and Section 3.2.

## 2. Multifunctional Photocatalytic Membranes: From Materials to Performance

### 2.1. Material Systems for Multifunctional Separation Membranes

#### 2.1.1. Metal Oxide-Based Membranes

The synergistic design of photocatalytic dye degradation and oil/water separation has evolved along two complementary directions: smart responsiveness and multifunctional integration. UV-responsive coatings enable light-triggered wettability switching and controllable separation-degradation operation [50], while MXene/ZnO heterointerfaces improve photocatalytic self-cleaning [51] and antibacterial/antiadhesion surface engineering strengthens multifunctional fouling control [38]. For more complex wastewater containing oils, dyes, and microorganisms, multifunctional integration introduces additional properties such as magnetism and anti-bioadhesion to enhance usability, durability, and comprehensive treatment capability [52,53,54]. These design strategies are not limited to TiO_2_ but are universally applicable to diverse photocatalytic systems, including CeO_2_, MnMoO_4_, BiOCl, and porphyrins, thereby broadening the spectral response range and enriching material selection [55,56,57,58,59,60]. For example, a MnMoO_4_-coated copper mesh rejects more than 99.9% of toluene-in-water emulsions and retains this separation efficiency even after abrasion and corrosion tests [56].

Precise nanostructural engineering enables TiO_2_-based membranes to achieve high-efficiency oil/water separation through controlled morphology and dimensionality. One-dimensional nanostructures, such as TiO_2_ nanofibers and nanorods, create micro/nano-rough surfaces with superhydrophilicity and underwater superoleophobicity, achieving approximately 99% separation efficiency for high-viscosity oils [43]. Viscous oil phases tend to adhere strongly to membrane surfaces and form thick, compact cake layers, which substantially reduces the permeate flux. Nevertheless, the permeate flux for highly viscous oils is generally lower than that for low-viscosity oils because of the higher hydraulic resistance of the viscous cake layer, and this trade-off should be carefully considered in practical process design. Zero-dimensional nanoparticles also remain effective when homogeneously dispersed in polymer matrices, as demonstrated by polypropylene (PP)/TiO_2_ melt-blown membranes, which deliver a high oil flux of about 15,000 L·m^−2^·h^−1^ while maintaining stable separation efficiency after repeated use [61]. Beyond these dimensional strategies, specific microscopic morphologies further refine separation performance. A mesoporous SiO_2_-TiO_2_ composite enables gravity-driven separation of both oil-in-water and water-in-oil emulsions; after 120 min of light irradiation, it degrades 99.3% of rhodamine B, 98.7% of methylene blue, 91.5% of methyl orange, and 96.2% of ciprofloxacin hydrochloride [62], while coaxial electrospinning produces core-sheath TiO_2_@ polyvinylidene fluoride (PVDF)/polyacrylonitrile (PAN) nanofibers with a maximum strength of 8.96 MPa and an ultrahigh oil flux of 40,163.79 L·m^−2^·h^−1^ sustained over 10 cycles [44]. Surface nano-coating offers another powerful approach: polydopamine (PDA)-coated TiO_2_ nanoparticles on filter paper maintain >99% separation efficiency over 17 cycles. The modified filter paper has a water contact angle of 28°, underwater oil contact angles above 150°, and completely photodegrades methylene blue under UV irradiation [63].

The wide bandgap of TiO_2_ (~3.2 eV for anatase) limits its photocatalytic activity to the ultraviolet region, posing a challenge for solar-driven wastewater treatment [64]. To address this, four main modification strategies have been developed. The first is heterojunction construction with narrow-bandgap semiconductors or noble metals, which extends light absorption and promotes charge separation. The second is ion doping with elements such as C, N, or W, N, which introduces intermediate energy levels within the bandgap [65,66]. The third is dye sensitization, which enables low-energy electron excitation through the insertion of dye highest occupied molecular orbital (HOMO) levels into the TiO_2_ bandgap [67]. The fourth is hybridization with carbonaceous materials such as modified carbon dots, which reduces the bandgap [68]. Collectively, these approaches tailor the optical and electronic properties of TiO_2_-based membranes for visible-light-driven applications.

#### 2.1.2. 2D Nanomaterial-Based Membranes

Graphene oxide (GO) nanosheets, derived from graphite oxidation and exfoliation, possess a 2D lamellar structure with abundant oxygen-containing functional groups (carboxyl, hydroxyl, and epoxy) on their basal planes and edges. These functional groups confer intrinsic hydrophilicity and negatively charged surfaces, enabling stable aqueous dispersion and the assembly of freestanding membranes through vacuum filtration or layer-by-layer deposition. They also serve as versatile anchoring sites for immobilizing functional species. The interlayer spacing of GO membranes is governed by hydration state and water intercalation, which can be tuned through external stimuli or chemical modifications, a property exploited for mass transport optimization.

The oxygen-containing functional groups on GO nanosheets (carboxyl, hydroxyl, and epoxy moieties) serve as highly efficient anchoring sites for the in situ growth or immobilization of diverse nanocatalysts. For instance, vacuum deposition of a TiO_2_/Co_3_O_4_/GO heterojunction coating on GO surfaces yields a membrane capable of photodegrading Congo red under simulated solar irradiation, while separating various oil/water emulsions with efficiencies exceeding 99.5%; both the underwater oil contact angle and the underoil water contact angle exceed 150°, and the performance is retained after 10 consecutive cycles [18]. Likewise, functional groups within GO interlayers act as nucleation sites for CoS_x_ nanoparticle growth. The resulting GO@CoS_x_ membrane achieves 99.84% methylene blue degradation upon peroxymonosulfate activation under alkaline conditions [69].

MXenes are a family of 2D transition metal carbides, nitrides, or carbonitrides with the general formula M_n+1_X_n_T_x_, where M is a transition metal, X is carbon and/or nitrogen, and T_x_ represents surface terminations such as -OH, -O, or -F. They exhibit metallic conductivity, hydrophilic surfaces, and excellent mechanical flexibility, making them promising candidates for multifunctional membranes in separation, catalysis, and energy conversion [14,24,28,49,70]. Their interlayer spacing can be tuned through intercalation strategies, while their surface properties can be further engineered via heterojunction construction or surface topography engineering.

MXene-based membrane systems have been extended to include photothermal conversion, resource recovery, and adsorption functionalities for the synergistic treatment of multi-component pollutants [71]. MXene-containing 3D aerogels can couple photothermal conversion with membrane separation and photocatalytic purification, as demonstrated by the aminated polyacrylonitrile (aPAN)/MXene@Ag-Ag_2_S system [72]. The carbon nanotubes (CNTs)-PAH/MXene membrane enables selective recovery of precious metal ions (Ag, Au, Pd) from oil/water emulsions with adsorption capacities of 527–631 mg/g. The recovered metals can be reused for catalysis and antibacterial applications, representing a shift from pollutant removal to resource valorization [73]. Additionally, amino functionalization imparts MXene membranes with high-efficiency dye adsorption and excellent aqueous stability, maintaining structural integrity and separation performance after 45 days of water immersion without the need for complex photocatalysts [74].

Visible-light-driven g-C_3_N_4_-based photocatalytic membranes can be constructed through morphological engineering, chemical pre-modification, or multifunctional integration. Li and colleagues fabricated self-standing membranes from seaweed-like g-C_3_N_4_ via vacuum self-assembly. The unique morphology imparts high surface roughness, large specific surface area, abundant transport channels, and intrinsic hydrophilicity, enabling a flux of 3114.0 L·m^−2^·h^−1^·bar^−1^ and 97.4% separation efficiency without substrates or binders. Its narrow bandgap also enables near-complete dye degradation and approximately 100% antibacterial efficiency, with stable performance over 35 cycles [75]. For composite membrane systems, chemical pre-modification addresses interfacial compatibility between g-C_3_N_4_ and polymer substrates. Khan and colleagues introduced polar functional groups via oxidation treatment to enhance g-C_3_N_4_-polypyrrole (PPy) interaction. The resulting membrane achieved >99% separation efficiency and recovered 96% of its flux after 30 min of solar light exposure [76]. Xue and colleagues immobilized hydroxyl-modified porous g-C_3_N_4_ nanosheets onto PVDF substrates using sodium alginate as an adhesive, achieving superhydrophilicity/underwater superoleophobicity with >99% oil rejection and photodegradation-based regeneration [77]. Beyond these strategies, g-C_3_N_4_ and its derivatives have emerged as versatile platform materials for diverse membrane processes, including reverse osmosis, nanofiltration, ultrafiltration, and oil/water separation, owing to their stability, environmental friendliness, and low cost, while also offering photocatalytic degradation and adsorption capabilities [78].

While the construction strategies discussed above enable g-C_3_N_4_ membrane fabrication, the intrinsic low quantum efficiency of g-C_3_N_4,_ caused by rapid electron–hole recombination, dense stacking, and single functionality, limits its photocatalytic performance [78]. Heterojunction construction with other semiconductors is the primary strategy to suppress recombination and prolong carrier lifetime. Alternative approaches include morphology engineering to increase surface area and active sites, and defect engineering to introduce mid-gap states that facilitate charge separation [75].

2D MOF nanosheets offer another route to precise oil/water sieving through wettability regulation. Surface modification with these nanosheets enables significant wettability control, achieving efficient and precise emulsion separation. Tian and colleagues assembled hydrophilic UiO-66-NH_2_ nanosheets onto hydrophobic polyphenylene sulfide substrates. The resulting micro/nano hierarchical structure transforms the surface from hydrophobic to superhydrophilic (complete water immersion in 0.6 s) and endows it with underwater superoleophobicity, achieving an oil contact angle of 163.1° for toluene. This membrane exhibits a high permeate flux of 7638.1 L·m^−2^·h^−1^ and 99.1% oil rejection for surfactant-free toluene-in-water emulsions, demonstrating the feasibility of 2D MOF nanosheet membranes for high-throughput, high-precision sieving [79].

#### 2.1.3. 3D Porous Framework Membranes

A design strategy based on 3D porous composite foams, constructed by integrating functional particles with a polymeric binder, offers an effective pathway to achieve high-flux oil/water separation under gravitational force, a critical advantage for the development of MOF-particle-based thick films or catalytic layers. This approach leverages the non-densely packed network formed by particle accumulation, which creates abundant micro- and nanoscale voids that substantially reduce hydraulic resistance while exposing a large fraction of particle surfaces for subsequent reactions. In a representative demonstration, Cui et al. fabricated a thermoplastic polyurethane (TPU)/TiO_2_/PDA 3D porous composite foam, wherein TiO_2_ nanoparticles were immobilized within a TPU matrix to form an open-cell structure. Remarkably, this foam achieved water and oil fluxes of 21,035 L·m^−2^·h^−1^ and 14,500 L·m^−2^·h^−1^, respectively, operating solely under gravity, while its separation efficiency for a petroleum ether/water mixture reached 99.6%. Extending this particle-packed thick film architecture to incorporate photocatalytic components enables synergistic integration of oil/water separation and organic dye degradation, simultaneously conferring self-cleaning properties that sustain long-term separation performance. Under ultraviolet light irradiation, the foam achieved a rhodamine B removal rate of 96% after only one hour, demonstrating the feasibility of concurrent dye degradation during separation operations. After ten consecutive cycles, the separation efficiency for petroleum ether/water mixtures remained as high as 98.5%, representing only a marginal decline from the initial 99.6% [45]. While these results underscore the durability imparted by the photocatalytic self-cleaning effect, two limitations merit consideration: first, the photocatalytic degradation data were obtained under UV light, leaving the performance under visible light or sunlight unclear; second, the cyclic tests evaluated separation efficiency but did not simultaneously track the photocatalytic activity across cycles. Nevertheless, the TPU/TiO_2_/PDA foam provides a compelling proof-of-concept that a particle-assembled, photocatalytically active thick film can achieve both high-rate dye degradation (96% in 1 h) and sustained separation performance (98.5% after 10 cycles), thereby establishing a reference benchmark for MOF-based thick films wherein the MOF itself either possesses intrinsic photocatalytic activity or serves as a host for embedded photocatalysts.

The construction of 3D macrostructures, such as foams and sponges, from low-dimensional building blocks is primarily achieved through two complementary routes: template-assisted freeze-drying and successive surface modification of commercial porous scaffolds [62,80]. In the freeze-drying approach, directional ice templating followed by sublimation yields a honeycomb-like interconnected porous network, and increasing the content of boron nitride nanofibers progressively shifts the foam surface from hydrophilic to hydrophobic [81]. In contrast, the surface modification strategy employs commercially available sponge skeletons for sequential deposition of functional components, thereby simplifying the fabrication of multifunctional 3D materials [82]. These 3D structures inherently possess high specific surface area, interconnected pore networks, and tunable surface wettability, collectively positioning them as ideal multifunctional platforms [5,81]. Integrating photocatalysts into 3D porous and flexible frameworks effectively overcomes the long-standing limitations of powdered catalysts, poor recoverability and inadequate mechanical stability, while enhancing photocatalytic degradation efficiency through increased surface area and enabling effective oil/water separation via engineered wettability [5]. In a representative demonstration, Sui et al. engineered a polyurethane-based sponge via integrated surface modification that exhibits both outstanding superhydrophobicity (with a maximum water contact angle of 161.64°) and photocatalytic activity. Furthermore, they designed a heterogeneous dual-functional system comprising an upper superhydrophobic layer for oil separation and a lower photocatalytic layer for dye degradation, achieving simultaneous oil/water separation and dye decomposition, with the sponge showing an oil absorption capacity of 60–109 g·g^−1^ [82]. The review by Sharma et al. further confirms that through engineered surface wettability combined with photocatalytic functionality, 3D sponges have emerged as a frontier platform for dual-function wastewater treatment. Collectively, these studies illustrate that whether through integrated modification or functional zoning, 3D macrostructures provide a versatile scaffold for integrating otherwise incompatible functions, with the design choice fundamentally representing a trade-off between fabrication simplicity and functional optimization [5,82].

**Figure 2:**
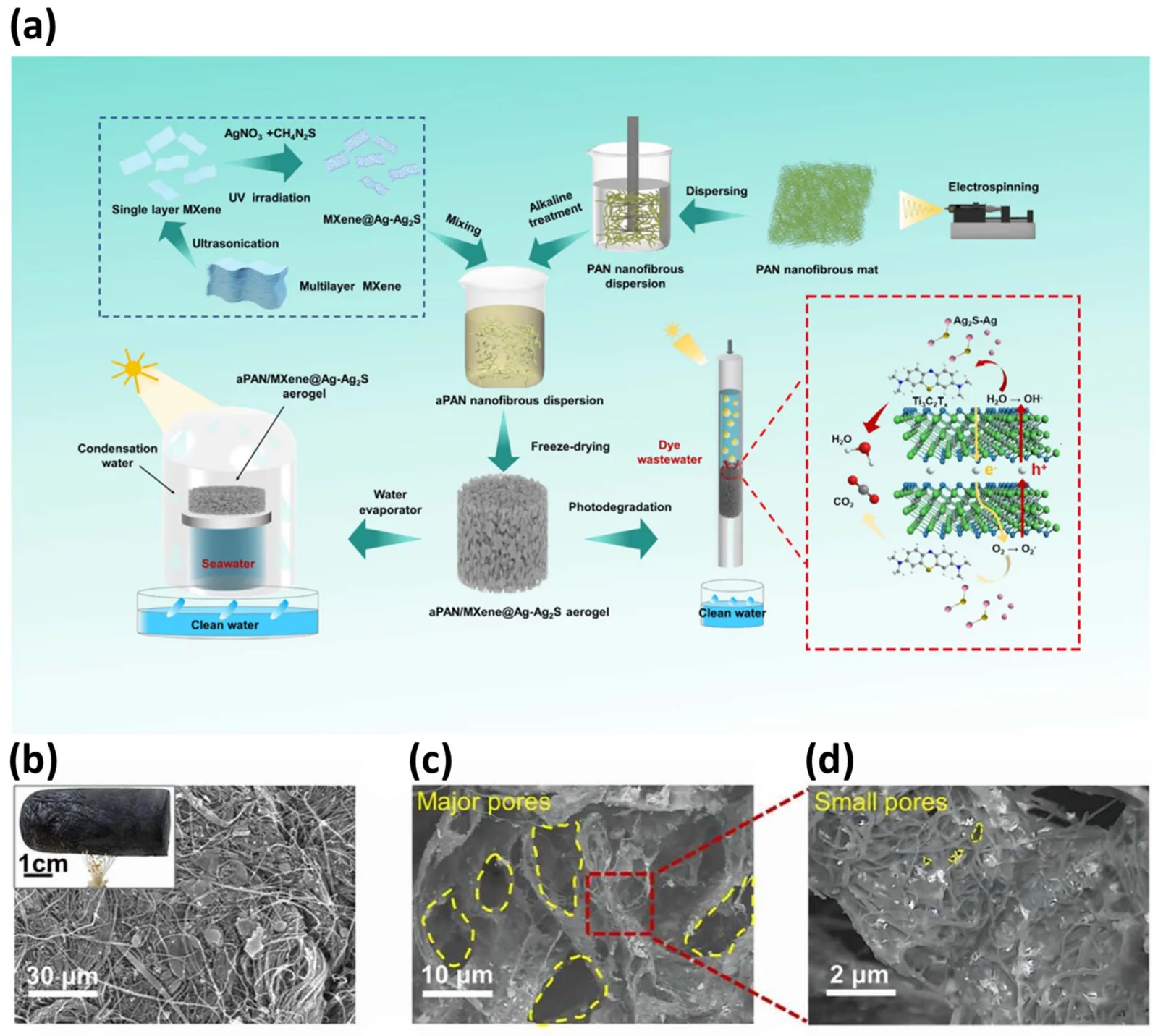
Fabrication and hierarchical pore structure of the aPAN/MXene@Ag–Ag_2_S nanofibrous aerogel. (**a**) Electrospinning and alkaline treatment of PAN nanofibers, UV-induced in situ growth of Ag–Ag_2_S on Ti_3_C_2_T_x_ MXene, freeze-drying, and coupled solar evaporation and photocatalytic degradation; (**b**) surface morphology, with the inset showing the monolithic aerogel; (**c**,**d**) cross-sectional scanning electron microscopy (SEM) images showing interconnected macropores and smaller pores at different magnifications. Reprinted from [72].

Assembling one-dimensional flexible nanofibers with 2D MXene nanosheets into 3D nanofibrous aerogels offers an effective strategy to overcome the inherent limitations of conventional 2D MXene membranes, including restricted specific surface area and incomplete photocatalytic degradation caused by densely packed structures. Wulayimujiang and colleagues demonstrated this approach by combining aPAN nanofibers with Ti_3_C_2_T_x_ MXene nanosheets, followed by UV light-induced in situ reduction of Ag-Ag_2_S nanoparticles (featuring two distinct structures) and freeze-drying, yielding the aPAN/MXene@Ag-Ag_2_S nanofibrous aerogel. The resulting material possesses an interconnected 3D porous network, in which aPAN nanofibers provide mechanical support, MXene nanosheets serve as photocatalytic and photothermal active sites, and the Ag-Ag_2_S heterostructure enhances visible-light absorption and charge separation (Figure 2a), collectively constructing a hierarchical pore structure (macro- and mesopores) that facilitates rapid fluid transport and intimate contact between pollutants and active sites (Figure 2b–d). This design directly addresses the critical bottleneck of conventional membranes in treating high-concentration dye wastewater: insufficient photodegradation arising from short residence time and high flux. By transitioning from 2D dense membranes to 3D macroporous aerogels, the strategy not only prolongs the interaction time between pollutants and functional sites but also integrates two complementary water treatment modes: filtration separation and solar interfacial evaporation. This paradigm shift in MXene-based membrane design provides a viable pathway toward one-step, high-efficiency purification of high-concentration dye wastewater [72].

The 3D porous network of hydrogels, serving as “nanoreactors” for photocatalyst immobilization, achieves synergistic optimization of mass transfer efficiency and catalytic activity through the rational design of pore connectivity, permeability, and surface wettability. Fu et al. employed a bubble-templating approach combined with in situ CO_2_ generation to fabricate calcium alginate gel beads featuring interconnected macropores, a structure explicitly described as “beneficial to the efficient mass transfer of pollutants,” which enabled the removal of approximately 99% of rhodamine B from water within 30 min under visible light irradiation [83]. However, the remarkable performance of this system relies on a multi-component pre-gel formulation and a two-step crosslinking/gas-generation process, potentially introducing formulation-dependent structural variability. In contrast, Vásquez et al. adopted cryopolymerization to construct poly(sodium acrylate) cryogels incorporating g-C_3_N_4_ nanosheets, yielding a 3D network with rapid water permeability and underwater superoleophobicity, thereby achieving gravity-driven filtration of oil/water-dye mixtures while circumventing the high energy demand of pressure-driven membrane processes, and exhibiting visible-light-excited photocatalytic self-cleaning capability [3]. Collectively, both strategies validate the core concept that 3D hydrogel networks can serve as efficient nanoreactors, immobilizing nanosheet photocatalysts within porous networks to maintain high permeability while enabling facile recovery. The complementarity of their design approaches, with one prioritizing structural interconnectivity for enhanced mass transfer and the other emphasizing low-energy gravity-driven operation integrated with self-cleaning, fully demonstrates the flexible tunability of hydrogel-based nanoreactors, while also underscoring that the optimal architecture remains contingent upon the specific operational context and the physicochemical properties of the target pollutants [84].

#### 2.1.4. Biomass-Derived and Green Membranes

**Figure 3:**
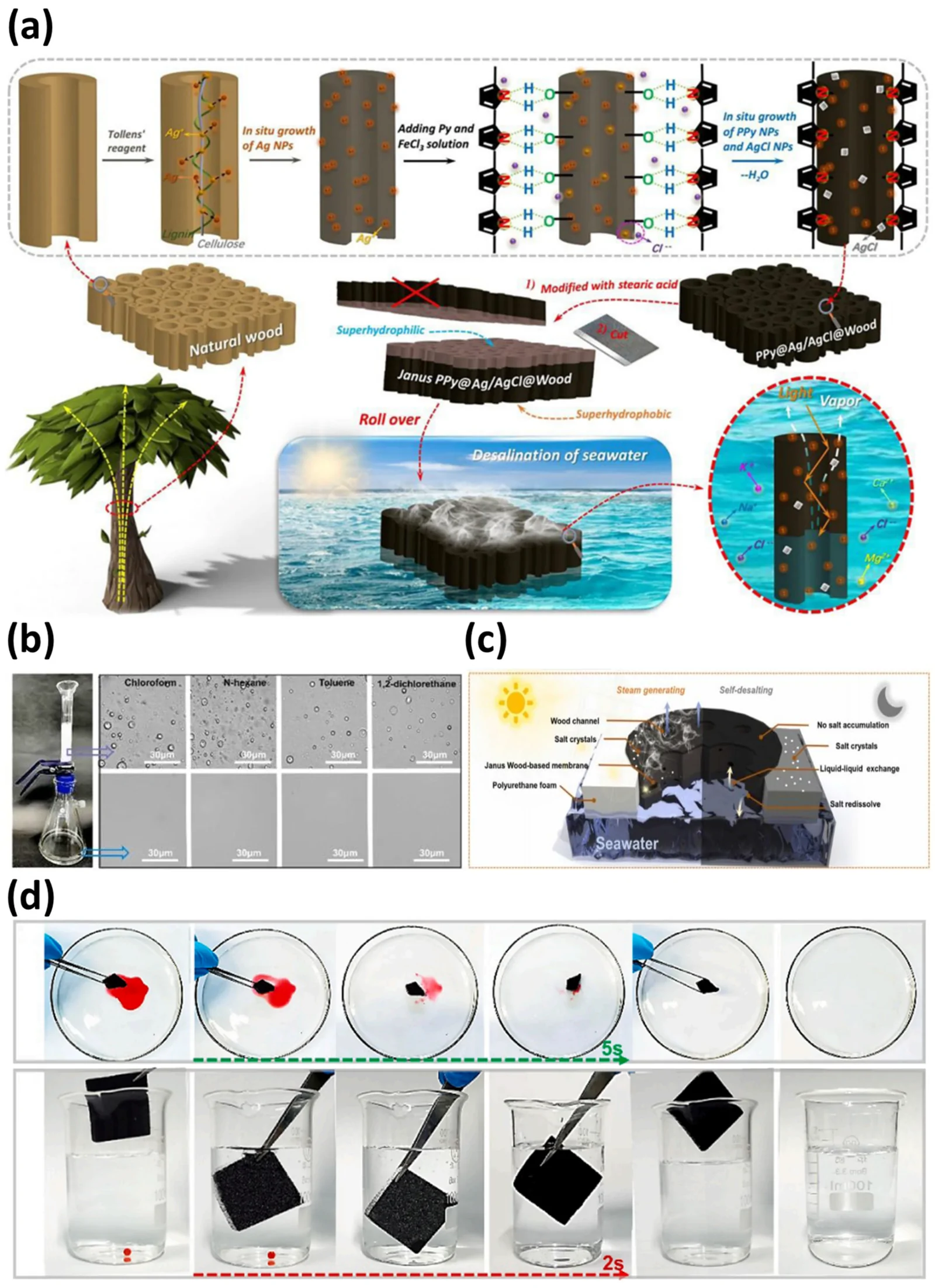
Construction and multifunctional operation of a Janus PPy@Ag/AgCl@wood membrane. (**a**) Fabrication of the mesoporous wood-based membrane; (**b**) oil/water separation and optical micrographs of emulsions before and after filtration; (**c**) steam-generation and self-desalting mechanisms; (**d**) oil adsorption above and below the water surface, with the membrane top face oriented upward. Reprinted from [35].

The inherent anisotropic porous architecture, 3D fibrous networks, and surface chemical characteristics of natural biomass materials provide a unique physical scaffold for constructing high-flux platforms for simultaneous oil/water separation and pollutant degradation. The longitudinally aligned microchannels of natural wood can be directly employed as gravity-driven filters, as exemplified by a ZnO-doped wood aerogel achieving a water flux of 39,400 L·m^−2^·h^−1^ with a separation efficiency exceeding 98.5%, along with photocatalytic degradation of up to 92.94% for methylene blue and an average oil adsorption capacity of 17.66 g·g^−1^ [85]. The porous structure of wood also serves as an ideal substrate for anchoring functional nanoparticles: Ag nanoparticle-modified balsa wood exhibited an underwater oil contact angle of 153°, achieving 94.4% methylene blue degradation and >99.97% inactivation rates against three bacterial strains [86]. Furthermore, a bioinspired Janus wood membrane exploiting natural transpiration-driven microchannels realized directional liquid transport without external energy input (Figure 3a), simultaneously accomplishing oil-in-water emulsion separation reaches 99.51% with the droplet population disappearing entirely from the post-filtration micrographs (Figure 3b), while top-surface photothermal evaporation delivers 95.33% solar conversion efficiency (Figure 3c), dye degradation (methylene blue 86.4%, rhodamine B 91.9%), and exhibits oil adsorption capability both above and below the water surface (Figure 3d) [35].

Natural cellulosic materials demonstrate equally remarkable value. TiO_2_-modified cotton fabric exhibited a water flux of 6800 L·m^−2^·h^−1^ with >99.90% separation efficiency [87], while two complementary cotton fabrics derived from a single preparation process enabled simultaneous efficient separation (>98.5% after 50 cycles) and dye degradation (90.1% for methyl blue, 95.2% for methyl orange) [8]. Bacterial cellulose membranes served a triple role as support, reducing agent, and stabilizer for the green synthesis of Pd nanoparticles, enabling concurrent emulsion removal and methylene blue reduction [59]. Nanocellulose carbonized derivatives, such as honeycomb-like carbon monoliths exhibiting superior performance to commercial activated carbon, further expand the application boundaries of cellulosic materials [88].

Certain agricultural wastes can be directly utilized without complex modification. Microfibers extracted from chestnut shells are naturally enriched with hydrocarbons, exhibiting a maximum oil absorption capacity of approximately 94% of their own weight and removal efficiencies of 91% for methylene blue and 88% for RhB [34]. The hollow microtubular architecture of kapok fiber serves as a “microreactor” for the green synthesis of Ag nanoparticles, achieving separation efficiencies of 99.90% for oil-in-water emulsions and 99.91% for water-in-oil emulsions [89]. Amyloid fibrils derived from protein waste also function as scaffolds for zeolitic imidazolate framework-8 (ZIF-8) biomimetic mineralization, effectively removing nine different heavy metal ions while exhibiting a dual adsorption–catalytic degradation pathway for synthetic dyes [90].

In summary, from the macroscopic anisotropic channels of wood to the multiscale fibrous networks of cellulose and the direct utilization of agricultural wastes, natural biomass materials, by virtue of their inherent structural advantages and surface chemical characteristics, offer highly promising green platforms for simultaneous oil/water separation and pollutant degradation. Nevertheless, specific performance metrics remain highly dependent on material source, modification strategy, and operating conditions, and the pursuit of an optimal balance between functionalization complexity and operational simplicity, scalability, and true waste-to-value conversion continues to be a central direction of ongoing research in this field.

The green functionalization of bio-based membranes fundamentally aims to replace toxic and non-degradable chemical reagents with natural, renewable, and biocompatible materials while employing energy-efficient, pollution-free processes, thereby endowing membranes with the dual capacity for oil/water separation and organic pollutant degradation [91]. Natural polyphenols, exemplified by dopamine, phytic acid (PA), and gallic acid (GA), have emerged as versatile green platforms due to their ability to undergo spontaneous deposition or metal ion coordination under mild aqueous conditions, with PDA modification introducing functional moieties that enhance pollutant binding, interfacial stability, and hydrophilicity while anchoring catalysts for degradation [91,92,93,94]. Similarly, PA-metal complexes and GA-derived coatings have enabled simultaneous rejection of emulsified oils and dyes with high efficiency [95,96], while eco-friendly composites combining ovalbumin, PA, egg shell powder, and TiO_2_ achieved gravity-driven separation with >99% efficiency and flux up to 13,458 L·m^−2^·h^−1^ [36]. As green alternatives to fluorinated agents, bio-derived low-surface-energy molecules such as stearic acid (STA) and natural polymers like chitosan have been widely employed, with STA decoration combined with various metal oxides/hydroxides consistently producing superhydrophobic/superoleophilic cellulose membranes exhibiting water contact angles of 162–166°, separation efficiencies of 88–98%, and photocatalytic dye degradation up to 98% [53,54,94,97,98]. For instance, the Co(OH)_2_/stearic-acid nanocellulose membrane degrades 93.66% of methylene blue and sustains oil/water separation fluxes of 87 L·m^−2^·h^−1^ with efficiencies above 93% even under strong acid/alkali conditions [98]. Physical processing methods, including dip-coating, spraying, layered filtration, electrospinning, and vacuum filtration combined with freeze-drying, offer a direct pathway toward “zero-chemical-pollution” functionalization, with notable examples including poly(lactic acid) (PLA)-polyethyleneimine (PEI) membranes achieving methyl orange adsorption capacity 78 times that of pristine PLA [99], nanocellulose/ZnO hybrid membranes prepared “without any chemical modification” exhibiting water permeance of 5439.7 L·m^−2^·h^−1^·bar^−1^ [100], and lignocellulosic nanofibril composites achieving 98.95% separation efficiency; the corresponding aerogel degrades 98.8% of dye under sunlight and adsorbs up to 37.6 g·g^−1^ of oil [101]. However, many physical methods still require subsequent chemical modifications to achieve superhydrophobicity, and performance metrics vary considerably [87,100,101]. For applications requiring enhanced structural stability, Schiff-base crosslinking offers a green alternative to conventional crosslinkers, as demonstrated by a cellulose–metal composite aerogel that retained 89% of its original strength after 10,000 compression cycles [102]. In practice, multifunctional bio-based membranes often integrate PDA, metal oxides and fatty-acid modification to combine emulsion separation, dye degradation and antifouling behavior within one platform [93,94,98]. Despite challenges including the life-cycle considerations of metal oxide nanoparticles and increased fabrication complexity, the judicious combination of naturally derived building blocks, mild processing techniques, and benign crosslinking chemistry represents the most promising pathway toward truly sustainable multifunctional membranes, preserving the inherent renewability and biodegradability of bio-based substrates while enabling the transition toward circular-economy-aligned wastewater treatment technologies.

The foundation of sustainable multifunctional membranes lies in a dual-source strategy that prioritizes renewable biomass over petroleum-based polymers while valorizing industrial solid wastes, thereby reducing dependence on virgin resources. PLA serves as a representative biodegradable substrate: a PLA@ZIF-8 composite membrane underwent almost complete degradation after 120 days of soil burial, avoiding white pollution, while achieving an oil/water separation efficiency > 99.8%, a flux of 6280 L·m^−2^·h^−1^, a diesel degradation rate of 92.8%, and a methylene blue removal rate of 99.7%; a PLA membrane modified with a superhydrophilic crosslinking network via hydrolytic co-deposition achieved an emulsion rejection rate > 99% and a cationic dye removal rate of 99.93% [46]; natural wood treated with deep eutectic solvent (DES) and decorated with palladium nanoparticles exhibited an oil/water separation efficiency of 98.9% and a methylene blue conversion rate of 99.8% [46]; and natural gallic acid (GA) was used to construct a GA-TiO_2_ coating on stereocomplex PLA membranes via aqueous-phase coordination bonding, yielding superhydrophilic and underwater superoleophobic properties without organic solvents [96]. Furthermore, industrial solid waste, fly ash, was utilized to fabricate a graphene oxide/geopolymer composite membrane, achieving a water flux of 1363 kg/(m^2^·h) and separation efficiencies ≥ 98.2% under gravity-driven conditions, while also exhibiting photocatalytic dye degradation under simulated solar-light irradiation. Regarding fabrication protocols, green strategies including seed-assisted in situ growth [103], hydrolytic co-deposition [104], and aqueous-phase coordination bonding [96] have been developed to eliminate toxic organic solvents; life-cycle assessment (LCA) quantitatively demonstrated that the one-step DES treatment introduces fewer environmental impacts than the traditional two-step pretreatment method. During the operational phase, the fly ash-based geopolymer membrane maintained separation efficiencies ≥ 98.2% in hot water, strong acid, strong alkali, and salt solutions [105], while the GA-TiO_2_ coated membrane retained its superhydrophilicity and underwater superoleophobicity in pH 1 solution and saturated NaCl; both the TiO_2_-containing PLA membrane [104] and the GA-TiO_2_ coated membrane [96] exploited the photocatalytic property of TiO_2_ under UV irradiation to decompose surface pollutants, achieving self-cleaning and extended service life; a geopolymer-armored copper mesh membrane, through ion exchange and chelation, reduced copper ion leaching concentration by two orders of magnitude, far below regulatory limits [106]. Finally, the biodegradability of the PLA@ZIF-8 membrane provides ultimate validation of sustainability [103], yet two additional PLA-based studies [96,103] did not provide experimental degradation data for their modified membranes, raising a critical question: whether the introduction of inorganic or hybrid functional layers alters the biodegradation kinetics of the PLA matrix, or whether these additives persist in the environment after polymer degradation. Nevertheless, the empirically proven biodegradability of PLA-based multifunctional membranes demonstrates that closing the material loop, from renewable feedstock to functional membrane to harmless degradation products, is an achievable goal, establishing a concrete pathway toward truly circular membrane technologies.

Table 2 maps the four representative material families of Section 2.1 onto their fabrication routes and operating modes. Two regularities emerge. First, driving force correlates with architecture rather than with chemistry, all 3D and wood-based systems in this review operate under gravity or capillary force, whereas every lamellar 2D membrane requires pressure. Second, in situ growth and vacuum filtration dominate the 2D family, while electrospinning dominates the fibrous family.

### 2.2. Membrane Structure and Interface Engineering Strategies

#### 2.2.1. Micro/Nano Structural Design and Wettability Control

The design of superhydrophilic/underwater superoleophobic surfaces for oil/water separation is fundamentally governed by the synergistic interplay between micro/nanoscale rough structures and hydrophilic surface chemistry [108,109,110]. Micro/nano rough structures amplify the intrinsic wetting behavior of a material: hydrophilic surfaces become superhydrophilic with hierarchical roughness, while hydrophobic surfaces become superhydrophobic. Representative implementations include agave-like Cu_3_Mo_2_O_9_ structures, β-FeOOH films, PPy nanowires, and porous glass-fiber supports [108,111,112,113]. For example, the agave-like Cu_3_Mo_2_O_9_-coated copper mesh reaches permeation fluxes of 3503.18 and 917.20 L·m^−2^·h^−1^ for surfactant-free and surfactant-stabilized oil-in-water emulsions, respectively, while photodegrading 92.4% of methylene blue and 68.6% of rhodamine B [104]. Surfactants stabilize oil-in-water emulsions by lowering the interfacial tension and forming protective interfacial films around the dispersed droplets, which suppresses droplet coalescence and aggravates membrane fouling via pore blockage. This pronounced flux decline is primarily attributed to the suppression of droplet coalescence and the formation of a more compact oil cake layer on the membrane surface, which increases the overall filtration resistance. These observations indicate that surfactant-stabilized systems represent a more stringent scenario for evaluating the practical performance of multifunctional separation membranes. Likewise, BiOBr-nanosheet-modified rock wool fibers continuously separate oil/water mixtures while removing more than 93.9% of rhodamine B by directly activating peroxymonosulfate [114]. Layered double hydroxide (LDH)-based sea-urchin and honeycomb surfaces likewise couple hierarchical texture with intrinsic hydrophilicity for separation and catalytic removal [115,116]. Catalytic surface chemistry has also been implemented on BiOBr-modified rock wool, TiO_2_-loaded PVDF fibers, and Fenton-active superhydrophobic meshes [114,117,118]. An ideal wetting state, characterized by a water contact angle approaching 0° and an underwater oil contact angle exceeding 150°, can be achieved through this design philosophy, yielding oil rejection rates frequently exceeding 99% and permeation fluxes ranging from thousands to tens of thousands of L·m^−2^·h^−1^ [1,15,108,111]. Biomimetic design translates natural wetting principles into engineered membranes, representative examples include nature-inspired MXene composites and fish-scale-inspired poly(N-isopropylacrylamide) (PNIPAM)/PAN/TiO_2_ nanofibers [14,119]. The fish-scale-inspired PNIPAM/PAN/TiO_2_ membrane shows underwater oil contact angles of 157° for petroleum ether and 151° for dichloromethane, decomposes 98% of rhodamine B under UV radiation, and its water flux switches from 10,013 ± 367 to 7713 ± 324 L·m^−2^·h^−1^ across the lower critical solution temperature [119]. Beyond static wettability control, smart membranes achieve reversible or on-demand separation through UV response, prewetting, electro-cleaning, and dual-superlyophobic surface design [50,112,115,120,121,122].

LDH-based membranes achieve superhydrophilicity and underwater superoleophobicity through the intrinsic synergy between their micro/nano hierarchical architecture and surface chemistry, without requiring post-synthetic modification [115,123]. The hydrothermal growth or grafting of LDH nanostructures onto porous substrates is the core strategy: Hou et al. grafted a honeycomb-like Co-Al LDH layer onto polyethylene terephthalate (PET) textiles [115], while Nian et al. achieved under-liquid dual superlyophobicity via in situ growth of interpenetrating Co-Al-LDH nanosheets on stainless steel mesh, suggesting that the optimal route may be substrate-dependent. This wettability enables exceptional separation performance: Nian et al. reported 99.7% efficiency for oil/water emulsions [123], and Wang et al. demonstrated a Co-Al-Ce LDH-coated mesh with an underwater oil contact angle of 158.5°, achieving >99.5% efficiency and a gravity-driven water flux of 52.2 L·m^−2^·s^−1^, far exceeding the pristine substrate (114.5°) [124]. The strategy is broadly applicable to ternary Co-Al-Ce LDH and ZnMgAl-LDH systems [124,125]. However, the incorporation of TiO_2_ nanoparticles in some LDH systems complicates the attribution of superwetting properties solely to the LDH architecture [125].

**Figure 4:**
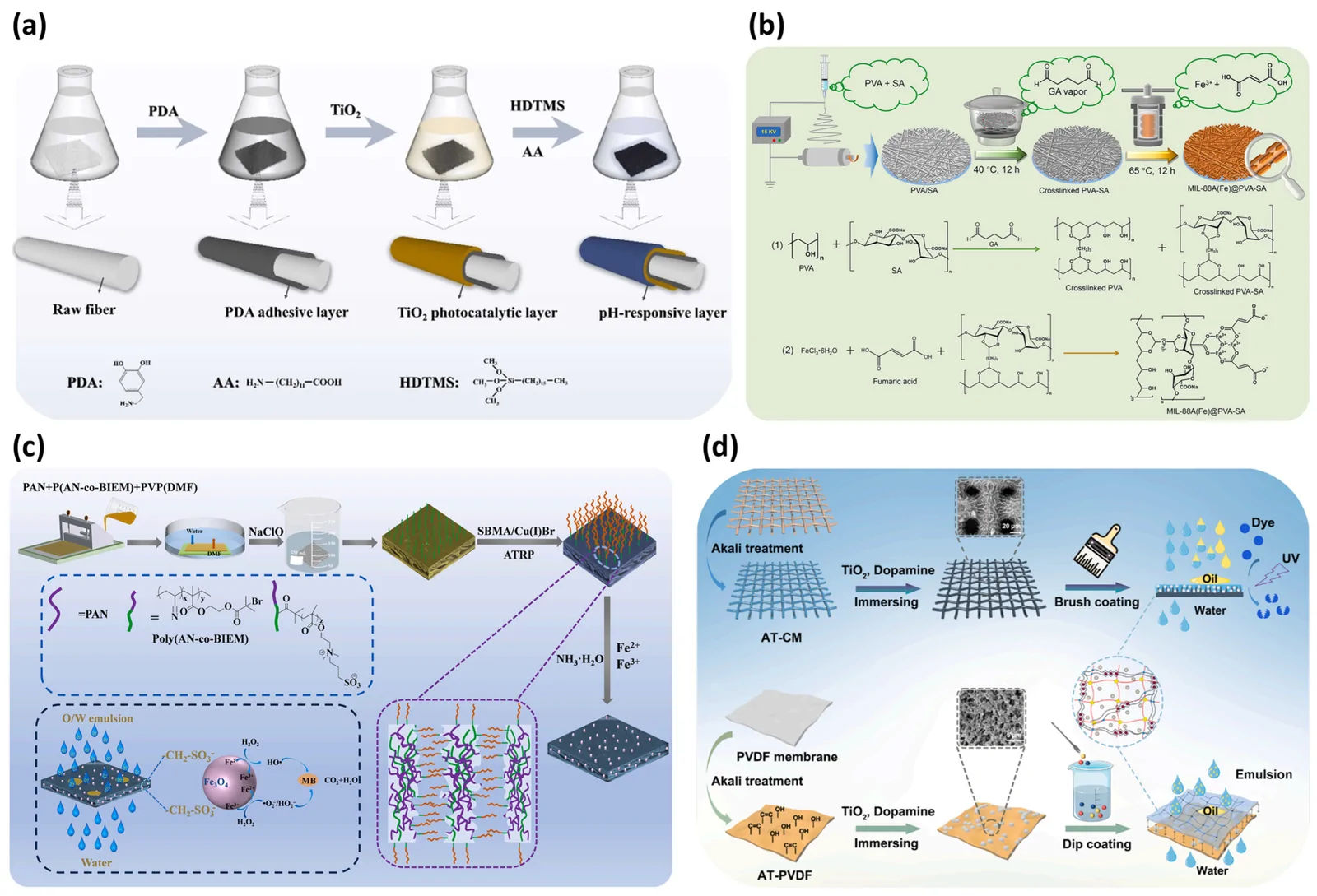
Layer-by-layer fabrication of functional composite membranes. (**a**) Sequential formation of the PDA adhesive, TiO_2_ photocatalytic, and pH-responsive layers; (**b**) preparation of the MIL-88A(Fe)@PVA–SA nanofiber membrane; (**c**) fabrication and surface modification of the Fe_3_O_4_@PAN–PSBMA-2 membrane for self-cleaning; (**d**) preparation of TiO_2_@PSA hydrogel-coated CM/PVDF. Reprinted from [65] for panel (**a**), [127] for panel (**b**), [126] for panel (**c**), and [128] for panel (**d**).

Hydration layers form through the stable binding of water molecules to surface functional groups of hydrogel materials, where the synergy between bound and surrounding water determines membrane wettability. Shao et al. grafted zwitterionic polymer brushes onto polyacrylonitrile membranes and demonstrated that bound and surrounding water in poly(sulfobetaine methacrylate) (PSBMA) enhance wettability [126], revealing a hierarchical structure of tightly bound inner water and dynamically exchanging outer water. A dense hydration layer manifests as superhydrophilicity (contact angle 0°) and underwater superoleophobicity (oil contact angle 161.7°), achieving separation efficiencies > 99.5% and fluxes of 1140–2455 L/m^2^/h. This extreme wettability prevents oil adhesion and enables regeneration through rinsing or photo-Fenton self-cleaning, with the MIL-88A(Fe)-decorated membrane degrading about 99.9% of methylene blue and reactive red 24 within 90 min under visible light [127]. However, the mechanical fragility of conventional hydrogel coatings limits their practical utility. To address this, Liu et al. developed a polyacrylamide-sodium alginate double-network hydrogel that maintained superwetting performance after abrasion and acid-base immersion, retaining 99.1% efficiency for copper mesh after 50 cycles and >99.3% for PVDF membranes [128]. The four routes in Figure 4 differ in the chemistry of the adhesion layer but converge on the same design logic. Two of them build the hydration layer from a polymer network PVA–SA nanofibers (Figure 4b) and a PSA hydrogel coating (Figure 4d), whereas the other two rely on an interfacial adhesive, PDA (Figure 4a), or on grafted zwitterionic brushes (Figure 4c). The distinction matters for durability rather than for wettability; network-based coatings retain performance after abrasion, whereas adhesive-anchored layers depend on the stability of a single interface, which is why Liu et al. turned to a double-network design [128]. Beyond passive hydration layers, stimuli-responsive wettability switching has emerged as an advanced strategy for on-demand oil/water separation. Documented switching routes include UV response, prewetting-induced tunability, electro-cleaning, and dual-superlyophobic surface design [50,112,115,120,121,122]. Janus double-layer fabrics, for example, feature asymmetric wettability design with a top superhydrophilic catalytic layer and a bottom superhydrophobic layer, enabling simultaneous oil/water separation and in situ degradation. This double-layer fabric achieves oil-removal fluxes above 2.7 L·m^−2^·s^−1^ with oil rejection exceeding 95%, while catalytically reducing aromatic dyes with efficiencies above 99.2% [17]. Electrically assisted and pH-responsive designs have both demonstrated reversible operation across repeated separation–cleaning cycles, although the reported cycle counts and test conditions differ among systems [65,120]. A representative HATP@CN membrane couples reversible pH-responsive wettability switching with bidirectional emulsion separation and UV-driven dye removal, thereby linking on-demand interfacial control with pollutant mineralization [65].

#### 2.2.2. Interlayer Engineering and Mass Transfer Channel Optimization

2D GO membranes face a fundamental permeability–selectivity trade-off: their tightly stacked lamellar structure enables precise solute rejection but imposes severe hydraulic resistance, rendering actual water flux orders of magnitude lower than theoretical predictions [1,29,129,130]. Intercalation strategies address this by introducing nanoscale spacers between GO nanosheets to expand the interlayer distance and create additional permeation pathways [1,29,129,130]. Zero-dimensional nanoparticle intercalation enables precise spacing control. Wei and colleagues expanded vermiculite nanosheet interlayer spacing from 1.352 ± 0.002 nm to 1.424 ± 0.004 nm via TiO_2_ nanoparticle intercalation, increasing water flux 14-fold to 261 L·m^−2^·h^−1^ while maintaining >98% dye rejection [130]. Ahmad and colleagues corroborated this using hierarchical CeO_2_/GO structures on nylon filters, achieving a pure water flux of 6451 L·m^−2^·h^−1^ and 99% oil rejection [1].

Beyond zero-dimensional approaches, intercalator morphology plays a decisive role in membrane homogeneity, channel regularity, and stability. Yu and colleagues showed that TiO_2_ nanowire-intercalated GO membranes achieve more uniform interlayer distribution, maintaining stable flux (273 L·m^−2^·h^−1^) and >98% removal over five cycles, outperforming nanoparticle-intercalated counterparts [129]. Chen and colleagues anchored TiO_2_ nanoparticles onto 3D crumpled GO spheres, creating a core–shell architecture with well-regulated water channels and permeance of 3142–3514 L·m^−2^·h^−1^ for oil-in-water emulsions (>99% rejection) [29].

The mechanistic framework underlying these enhancements is tripartite: physical interlayer expansion (14-fold flux increase) [130]; additional nanochannel formation through intercalator-GO interfacial regions and inter-particle voids (CeO_2_/GO: 6451 L·m^−2^·h^−1^) [1]; and enhanced surface hydrophilicity from CeO_2_ nanoparticles, TiO_2_/dopamine synergy, or uniform nanowire distribution. Critically, rejection rates remain >98–99% across all studies, demonstrating that permeability gains do not compromise the size-exclusion functionality of 2D membranes [1,29,129,130].

MXene-based membranes also benefit from interlayer engineering to overcome the permeability–selectivity trade-off. Wang and colleagues used biomass-derived chitin nanocrystals coated with TiO_2_ (ChNC@TiO_2_) as green intercalants, nearly doubling pure water permeance from 2450 to 4480 L·m^−2^·h^−1^·bar^−1^ through unique water channel formation [131]. An alternative, more radical approach, structural reconstruction, avoids compact stacking altogether. Duan and colleagues constructed a 2D/2D MXene/porous ZnIn_2_S_4_ heterojunction with a porous petal-like architecture, achieving an ultrahigh permeability of 5471.13–6732.57 L·m^−2^·h^−1^ [132].

#### 2.2.3. Heterojunction Construction and Band Engineering

TiO_2_-based heterojunctions have been widely developed for simultaneous oil/water separation and pollutant degradation. Representative TiO_2_-containing systems, from eco-friendly ovalbumin/eggshell-powder composites to BiOI/TiO_2_ heterojunctions, combine oil/water separation with light-assisted removal of dissolved organics. For the BiOI/TiO_2_ heterojunction, visible-light degradation reaches 85.62% for crude oil and 38.30% for 4-chlorophenol, compared with 70.56% and 0.0% over pristine TiO_2_ [36,133]. Ag/TiO_2_-based fibrous and carbon-cloth membranes further demonstrate dye degradation, photocatalytic self-cleaning and high cyclic separation stability. For the carbon-cloth membrane, the underwater oil contact angle reaches 156° and the separation efficiency 99.76%; visible-light self-cleaning nearly restores the performance within 30 min, and an efficiency of 99.65% is retained after 100 cycles [134,135]. Together with mechanistic reviews of TiO_2_ photocatalytic membranes, these examples support heterojunction and metal–semiconductor interface engineering as practical routes for improving light utilization and charge carrier separation [136,137].

Leveraging the metallic conductivity and hydrophilic surfaces of MXenes, heterojunction construction with semiconductors such as ZnO and Cu_2_O offers a versatile platform for multifunctional photocatalytic membranes. Systems including Ag@MXene, N-doped Bi_2_O_2_CO_3_/poly(arylene ether nitrile) (PEN), and MXene/ZnO simultaneously endow membrane materials with excellent photocatalytic self-cleaning capabilities (e.g., 95.24% degradation of methyl orange and 95.61% of crystal violet within 60 min) and antibacterial performance (e.g., approximately 99.99% inhibition of *E. coli*), thereby achieving synergistic antifouling effects and significantly enhancing long-term operational stability. To further overcome the bottleneck of photocatalytic efficiency, heterojunction and Schottky junction engineering have been introduced, leveraging intimate interfacial contact and matched energy level alignment between MXene and semiconductors to promote efficient separation and migration of photogenerated charge carriers. This approach enhances the degradation efficiency of composite membranes toward dyes like congo red by factors of 2.7 to 7.4 compared to single-component membranes, while maintaining oil/water separation efficiency exceeding 99.4% [15,80,107].

**Figure 5:**
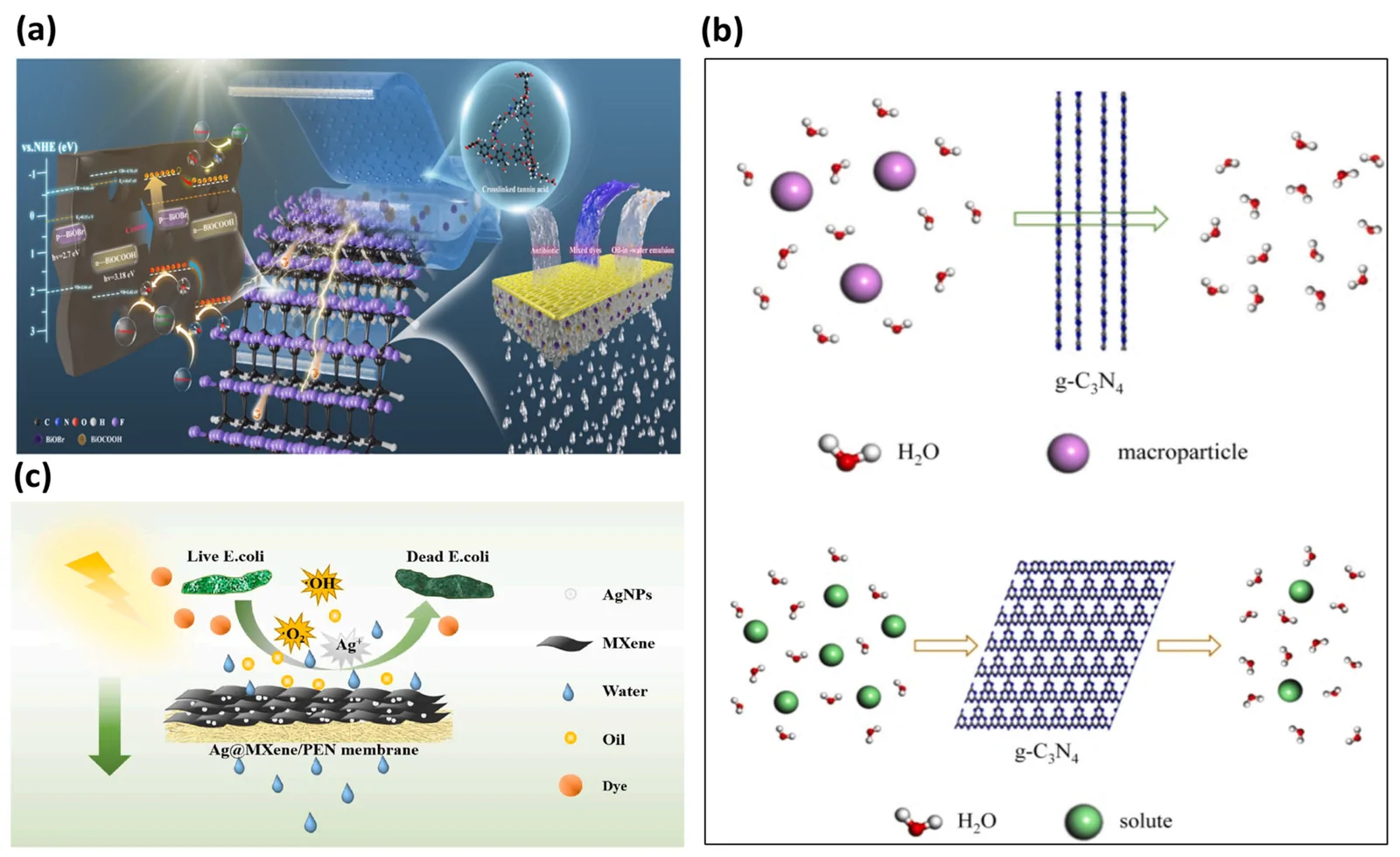
Membrane architectures and mechanisms supporting coupled separation, photocatalysis, and antifouling. (**a**) p–n BiOBr/BiOCOOH heterojunction membrane showing interfacial charge transfer and concurrent removal of antibiotics, dyes, and oil-in-water emulsions; (**b**) g-C_3_N_4_-based particle-entrapment and solvent-dehydration mechanisms; (**c**) separation and antifouling mechanism of the Ag@MXene/PEN fibrous composite membrane. Reprinted from [138] for panel (**a**), [78] for panel (**b**), and [107] for panel (**c**).

The photocatalytic performance of g-C_3_N_4_-based membranes is constrained by intrinsically low quantum efficiency, caused by rapid electron–hole recombination, dense stacking, and single functionality. Heterojunction construction with other semiconductors offers a direct route to suppress recombination and prolong carrier lifetime by forming an interface with matched band alignment. Among various designs, the 2D/3D heterojunction, exemplified by intercalating 3D flower-like TiO_2_@MnO_2_ nanostructures as electron acceptors into 2D g-C_3_N_4_ nanosheets, achieves spatial separation of electrons and holes, narrowing the bandgap to 2.10 eV and broadening light absorption, resulting in approximately 100% degradation of methylene blue and malachite green as well as approximately 100% photocatalytic antibacterial efficiency; the same membrane separates five oil-in-water emulsions with a maximum flux of 3265.67 ± 15.01 L·m^−2^·h^−1^·bar^−1^ and rejects 99.69% ± 0.45% of toluene-in-water droplets [54,78]. Complementing this mechanism, the p-n heterojunction leverages the built-in electric field at the interface to drive carrier separation and transfer. Chen and colleagues constructed a BiOBr-BiOCOOH p-n heterojunction embedded within a PVDF membrane, achieving average rejection rates of 95% for dyes and 86% for antibiotics, with photodegradation rates exceeding 99% for various pollutants and surpassing 98% even for mixed-dye wastewater. Whether achieved through 2D/3D interface engineering or p-n junction formation, the unifying principle lies in suppressing photogenerated carrier recombination via band engineering and built-in electric fields, a paradigm that defines the design of high-efficiency g-C_3_N_4_-based photocatalytic membranes [54,78,138]. Figure 5 places three charge-separation mechanisms side by side. In the p–n BiOBr/BiOCOOH membrane, separation is driven by the built-in field at the junction (Figure 5a); in the g-C_3_N_4_ system, the dominant contribution is geometric, particle entrapment and solvent dehydration within the interlayer (Figure 5b); in Ag@MXene/PEN, it is the Schottky barrier at the metal–semiconductor contact (Figure 5c). Comparing the three clarifies that ‘heterojunction’ in this literature denotes at least three physically distinct mechanisms.

In addition to conventional heterojunctions, quantum dots provide a distinct band engineering route through size-dependent quantum confinement effects. Reducing material dimensions below the exciton Bohr radius discretizes energy bands and widens the bandgap, offering a tunable platform for band engineering. Liang et al. synthesized Ag@AgCl quantum dots (10–20 nm) via oil-in-water self-assembly and decorated them onto flower-like Bi_2_WO_6_ surfaces. The excellent UV-vis absorption of this composite arises from the synergistic effects of quantum confinement, surface plasmon resonance of Ag nanoparticles, and the unique flower-like architecture of Bi_2_WO_6_. Photoelectrochemical measurements confirmed that suitable band alignment between Ag@AgCl and Bi_2_WO_6_, combined with metallic Ag, enables highly efficient charge separation. Consequently, the optimal Ag@AgCl (20 wt.%)/Bi_2_WO_6_ sample achieved 97.61% Rhodamine B degradation within 2 h under visible light, 1.33 and 1.32 times higher than pure Ag@AgCl and pure Bi_2_WO_6_, respectively [139]. A similar principle applies to non-quantum-dot systems: Ji et al. fabricated side-by-side electrospun nanofibers with ZnO on one side and Ag on the other. This ordered nanostructure achieved 97% dye degradation within 140 min, substantially outperforming the 80% degradation of randomly mixed single nanofibers. The critical determinant is the rational design of charge separation pathways rather than strict adherence to quantum confinement regimes perse [140].

#### 2.2.4. Surface Functionalization and Active Site Construction

Surface functionalization and active site construction are essential for endowing separation membranes with catalytic capabilities. The core strategy lies in precisely engineering surface functional groups, such as amino-rich moieties or carboxylate-containing crosslinking networks, to construct membrane surfaces with specific charges or chemical affinities, enabling selective and efficient enrichment of target pollutants. This enrichment mechanism operates through multiple physicochemical interactions, including electrostatic attraction, coordination, and hydrogen bonding, which preconcentrate pollutants in close proximity to catalytically active sites. The architectural compatibility, the ability to combine enrichment functionality with catalytic activity within a single membrane construct, constitutes a critical design principle. This section reviews three representative strategies for surface functionalization and active site construction: (1) catalytic functionalization of MOF membranes via Fenton-like reactions and integrated systems; (2) covalent organic framework (COF) functionalized pores as nanoreactors for confined catalysis; and (3) redox catalytic centers (MnO_2_/Ag) for Fenton-like oxidation and self-cleaning.

The core concept of catalytically functionalized MOF membranes lies in synergistically integrating the catalytic properties of MOFs with membrane separation processes, enabling simultaneous physical separation and chemical degradation on a single platform. Iron-based and bimetallic MOF materials inherently possess dual functionality: superwetting surfaces for oil/water separation and catalytic active centers for pollutant degradation. Fe-BTC films, for instance, exhibit a water contact angle exceeding 154° while achieving photodegradation rates of 87% for tetracycline and 97% for rhodamine B [141]; bimetallic LiSn-MOF combines superhydrophobic surfaces with visible-light photocatalytic activity (bandgap: 2.96–3.14 eV) [142]; and Fe-O clusters in Fe-MOF enable oil removal via adsorption while achieving over 90% dye degradation through photo-Fenton-like reactions [118].

Beyond photocatalysis, heterogeneous Fenton-like reactions based on transition metal nodes (Co, Fe) within MOFs offer a powerful alternative through peroxymonosulfate (PMS) activation to generate reactive radicals. A Co/Fe bimetallic MOF membrane achieved >99.74% methylene blue degradation within 40 min via PMS activation, with sulfate radical identified as the dominant reactive species [143]; CoNi-ZIF@PAN membrane with prewetting-induced reversible wettability enabled switchable separation of oil-in-water (flux > 1598 L·m^−2^·h^−1^, 97.04% efficiency) and water-in-oil emulsions (flux > 1127 L·m^−2^·h^−1^, 99.96% efficiency) while achieving 99.38% methylene blue degradation within 20 min [121]; PAN@Co-MOF membrane achieved 99.3% methylene blue degradation within just 10 min [122]; and a MIL-88A-based membrane specifically targeting “colored emulsions” degraded adsorbed dyes via photo-Fenton reaction while maintaining a flux close to 5000 L·m^−2^·h^−1^·bar^−1^ across 10 cycles; before cycling, the flux reaches up to 5062.7 ± 189.4 L·m^−2^·h^−1^ at a separation efficiency of 99.11 ± 0.18% [144].

To overcome the limitation of low catalyst-pollutant collision frequency, MOF membranes frequently employ an “adsorption-enrichment followed by catalytic degradation” synergy. A porous ZIF-8@PAN membrane achieved >90% organic dye removal through the synergistic effect between electrostatic adsorption and ZIF-8 photodegradation [145]; PCN-224/TA/PVDF membrane similarly removed water-soluble dyes through both adsorption and photocatalysis [146]; and the MIL-88A membrane first removed 99% of dyes through adsorption, then degraded them via visible-light-driven photo-Fenton reaction, creating a self-sustaining cycle [144]. To further enhance quantum efficiency, heterojunction and band engineering strategies have been introduced. In situ growth of seaweed-like ZnIn_2_S_4_ on ZIF-8@PAN membrane created a well-defined heterojunction interface. Density functional theory (DFT) calculations and free radical inhibition experiments confirmed that this structure facilitated photo-induced charge carrier transfer and separation, achieving 96.88% photocatalytic degradation efficiency for various dyes under visible light while maintaining a flux of 4408.1 L·m^−2^·h^−1^ after 10 cycles [147].

Current research is progressing toward highly integrated systems. CoNi-ZIF@PAN and PAN@Co-MOF membranes combine PMS catalytic activity with switchable superwettability, creating intelligent platforms for “on-demand separation plus efficient catalysis” [121,122]; Cu-doped UiO-66-NH_2_ simultaneously enhanced hydrophilicity and visible-light photocatalytic performance, maintaining 99.7% separation efficiency and 99.5% dye degradation rate under extreme conditions including strong acids/bases and abrasion [148]; NH_2_-MIL-125 demonstrated the synergistic effect of ligand and metal cluster engineering [149]; and a CNTs-PAA/MIL101(Fe)@Pt composite membrane achieved an ultrahigh separation throughput of 11,000 L·m^−2^·h^−1^·bar^−1^, addressing the engineering challenge of reconciling high flux with multifunctionality [150].

COFs offer ordered nanochannels that can be engineered into active “functional nanoreactors” for environmental remediation. The core strategy is to use COFs as high-surface-area supports for immobilizing catalytic species, overcoming the practical limitations of nanomaterial powders. In the Pd@COF/wood membrane, for example, palladium nanoparticles confined within COF pores achieve reduction reaction rate constants of 0.72 min^−1^ for 4-nitrophenol and 0.85 min^−1^ for methylene blue. This confinement effect not only prevents nanoparticle aggregation but also creates nanoscale reaction environments that facilitate substrate access to active sites. When operated as a filter membrane, this system achieves >98% methylene blue degradation while maintaining a permeance of 1.57 × 10^4^ L·m^−2^·h^−1^·bar^−1^, demonstrating that functionalized pore design enables efficient degradation without compromising high throughput. The membrane also exhibits exceptional recyclability and oil/water separation ability, confirming that the benefits of pore functionalization extend to broader remediation functionalities. The realization of such synergistic performance without sacrificing membrane integrity underscores the core value of engineering COF channels as nanoreactors rather than passive conduits [151].

Metal-based catalytic redox centers provide an effective strategy for achieving simultaneous oil/water separation and organic pollutant degradation by activating peroxymonosulfate (PMS) through a Fenton-like pathway to generate strong oxidizing radicals. Zheng et al. fabricated a Janus nylon membrane with in situ generated silver nanoparticles, while Zhang et al. constructed a MnO_2_ mineralization coating on a PVDF nanofiber membrane; both approaches transform the membrane surface into an efficient platform featuring metal catalytic centers. The PVDF@TA/MnO_2_ membrane achieved 96.5% removal of methylene blue in the presence of PMS, with electron spin resonance spectroscopy confirming the generation of strong oxidizing radicals during the catalytic process [152]. Although the Ag-based Janus membrane did not employ PMS, it still achieved a dye degradation efficiency exceeding 97.3%, indicating that different metal centers can achieve comparable catalytic performance through distinct activation pathways. More importantly, these redox centers couple catalytic degradation with membrane separation: the MnO_2_/PMS system mitigates membrane fouling through self-cleaning action, while the Ag-decorated Janus membrane exhibits heavy metal ion adsorption capacity concurrently with emulsion separation [153]. Thus, redox centers based on MnO_2_ or Ag simultaneously achieve oxidative degradation of organic pollutants, membrane fouling mitigation, and heavy metal adsorption, offering an ideal platform for treating complex wastewater containing both emulsified oils and dissolved contaminants.

### 2.3. Separation–Catalysis Synergistic Mechanisms and Multifunctional Integration

The theoretical foundation of photocatalysis–separation synergy originates from the unique photo-responsive properties of titanium dioxide (TiO_2_): the photogenerated reactive oxygen species drive the photocatalytic mineralization of pollutants, while the photo-induced superhydrophilicity provides the necessary conditions for constructing underwater superoleophobic surfaces, both stemming from a common photophysical mechanism [37]. Building upon this theoretical basis, loading TiO_2_ onto porous substrates achieves integration of separation and catalysis by tuning surface wettability, yielding separation efficiencies exceeding 98% and dye degradation rates above 90% across diverse substrates including electrospun fibrous membranes, metal meshes, fabrics, and microspheres [116,119,128,154,155,156,157]. The heterojunction and composite strategies for TiO_2_-based systems are detailed in Section 2.1.1, and their synergistic mechanisms are further elaborated below.

MXene-based heterojunctions exhibit synergistic photocatalytic self-cleaning and antibacterial effects through efficient charge separation at the heterointerface. This synergy significantly enhances long-term operational stability by preventing both organic fouling and biofouling, achieving dye degradation rates approximately 2.7 to 7.4 times higher than those of single-component membranes while maintaining oil/water separation efficiency above 99.4% [15,80,107].

For MOF-based systems, incorporating photoactive MOFs into membrane architectures enables simultaneous oil/water separation and degradation of dissolved dyes. A MIL-53(Fe)-intercalated MXene composite membrane achieved oil-in-water emulsion retention rates of 99.06–99.62% and methyl blue photodegradation of 99.0% [158]; porphyrinic Zr-MOF (PCN-224) with photosensitive porphyrin ligands exhibited visible-light responsiveness and removed water-soluble dyes through an adsorption–photocatalysis synergy with separation efficiency > 99% [146]; amine-functionalized Ti-MOF (NH_2_-MIL-125) maintained >99% removal efficiency for both emulsions and dyes after 10 cycles [149]; Mil-53(Fe)/PAN membrane achieved 100% dye degradation under visible light with persulfate [159]; and a ZIF-8-based superhydrophobic membrane extended applicability to water-in-oil emulsion separation (efficiency > 99.9%) while retaining photocatalytic degradation and antibacterial functions [160].

Extending the photocatalytic functionality to 3D architectures, 3D hydrogel networks serving as “nanoreactors” for photocatalyst immobilization achieve synergistic optimization of mass transfer efficiency and catalytic activity through the rational design of pore connectivity, permeability, and surface wettability. Calcium alginate gel beads featuring interconnected macropores enabled the removal of approximately 99% of rhodamine B from water within 30 min under visible light irradiation [83], while poly(sodium acrylate) cryogels incorporating g-C_3_N_4_ nanosheets achieved gravity-driven filtration of oil/water-dye mixtures while exhibiting visible-light-excited photocatalytic self-cleaning capability [3]. These two approaches, one prioritizing structural interconnectivity and the other emphasizing low-energy gravity-driven operation, demonstrate the tunability of photocatalysis–separation synergy, while underscoring that the optimal architecture depends on the specific operational context and target pollutants.

**Figure 6:**
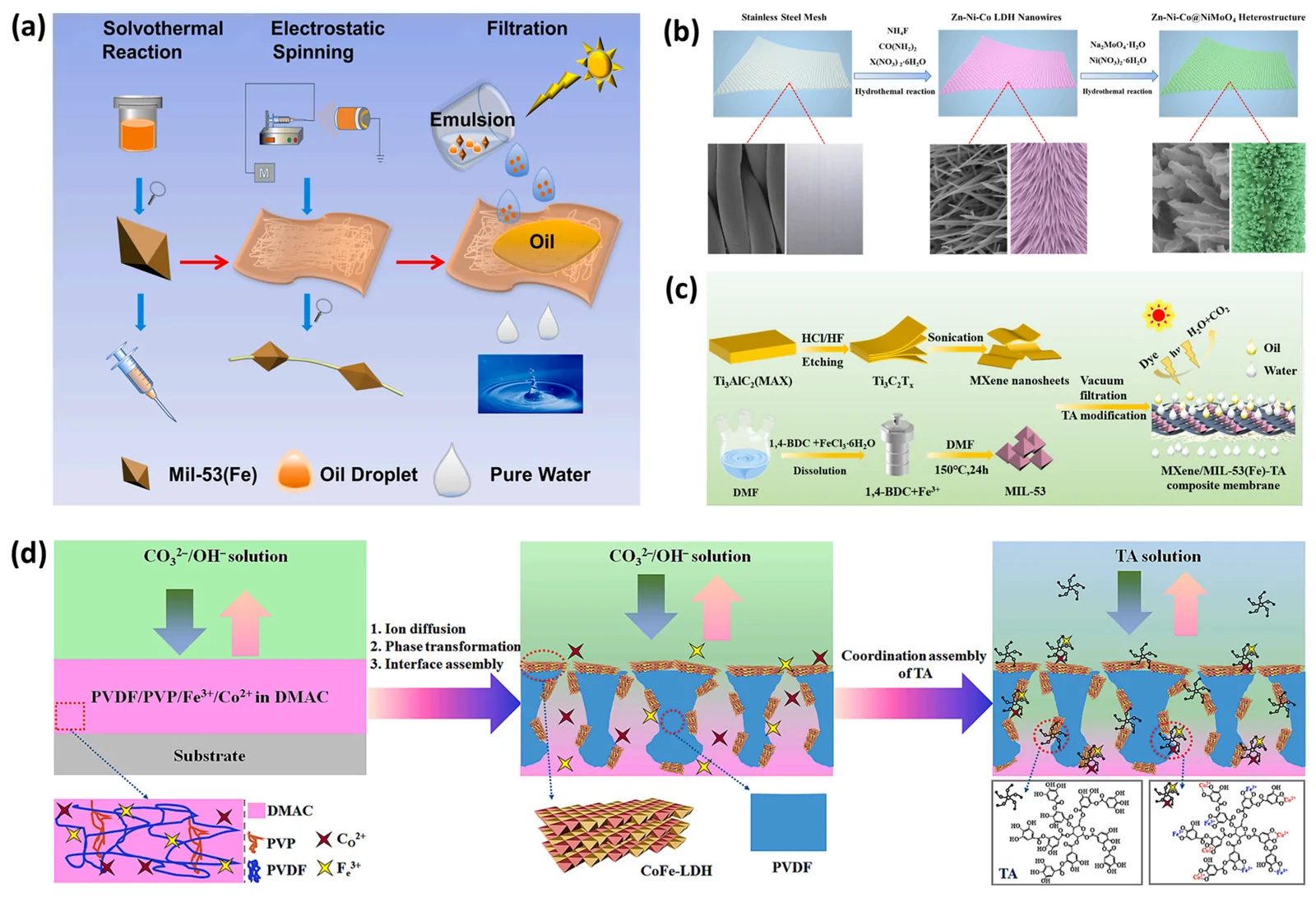
Multistep assembly of functional membranes. (**a**) Membrane fabrication and emulsion separation; (**b**) preparation of ZNC@NM–SSM, using Co(NO_3_)_2_·6H_2_O, Ni(NO_3_)_2_·6H_2_O, and Zn(NO_3_)_2_·6H_2_O precursors; (**c**) preparation of MXene nanosheets and MIL-53(Fe), followed by vacuum filtration and tannic acid (TA) modification; (**d**) preparation of the TA@CoFe-LDH@PVDF membrane. Reprinted from [159] for panel (**a**), [163] for panel (**b**), [158] for panel (**c**), and [161] for panel (**d**).

LDH-based membranes function as highly efficient heterogeneous advanced oxidation process (AOPs) catalysts, enabling simultaneous oil separation and degradation of dissolved organic pollutants. This dual functionality arises from the well-dispersed catalytic active sites inherent to LDH structures, which can initiate AOPs to eliminate various water-soluble organic pollutants including pharmaceuticals, phenolic compounds, and organic dyes [116,161,162]. Liu and colleagues demonstrated that tannic acid-mediated CoFe-LDH hybridized PVDF membranes achieved nearly 100% degradation of methylene blue within 5.0 min via peroxymonosulfate (PMS) activation, with a rate constant of 1.04 min^−1^, 2.67 times higher than that of unmodified CoFe-LDH@PVDF membranes, while the assembled tannic acid layer also inhibited metal ion leaching [161]. Beyond PMS-activated chemical oxidation, LDH-based membranes exhibit remarkable tunability toward visible-light-driven photocatalysis. Zn-Ni-Co LDH@NiMoO_4_ heterojunction nanoarrays with visible light responsiveness were constructed for efficient dye degradation [163], with the coated mesh showing an underwater oil contact angle of 164.9° and retaining separation efficiencies above 99.90% after 60 cycles, while PPy integrated with CoZn-LDH promoted photogenerated electron–hole separation, achieving 84.1% photocatalytic degradation of tetracycline hydrochloride under neutral pH, along with effective degradation of rhodamine B, methylene blue, and methyl orange [67]. The most transformative advantage lies in dynamic filtration processes, where forced convective mass transfer dramatically accelerates catalytic kinetics, the degradation rate under dead-end filtration was 23.89 times higher than under static conditions [161]. The four assemblies in Figure 6 share a common feature that explains this acceleration: in every case the catalytic layer is placed on the feed-facing surface (Figure 6a–d) rather than embedded in the bulk, so that convective flux through the membrane continuously renews the foulant concentration at the active sites. This is the structural reason why the degradation rate under dead-end filtration exceeds the static value by a factor of 23.89 [161].

The Fenton-like activity of MOF nodes toward PMS, together with the switchable-wettability platforms it enables, has been described in Section 2.2.4; from the standpoint of separation–catalysis synergy, its distinguishing feature is that radical generation and emulsion demulsification are triggered by the same transition-metal center. The practical viability of these membranes is further supported by their broad-spectrum degradation capability and robust stability: urchin-like Ni/Co LDH stainless steel mesh exhibited excellent physical, chemical, and thermal stability in acidic, alkaline, and saline environments [116], and LDH-modified activated carbon fibers maintained exceptional removal rates for both oil and organic pollutants during continuous filtration of complex sewage [162].

Furthermore, the MnO_2_- and Ag-based redox centers described in Section 2.2.4 illustrate a second form of coupling: the same surface that activates PMS also adsorbs heavy metal ions, so that oxidative degradation, fouling mitigation and metal capture proceed on a single interface rather than in sequence [152,153].

Beyond these synthetic platforms, the transition of Prussian blue analog (PBA)-based membranes from a single catalytic function toward integrated separation and adsorption capabilities hinges critically on nanoscale architectural design. The construction of 3D FeCo-PBA@GO functional microspheres overcomes the low porosity and poor antifouling performance of conventional graphene oxide membranes caused by dense nanosheet stacking. This unique 3D nanostructure prevents nanosheet stacking, enhances oil repellence, regulates water channels, and breaks through permeability limitations, thereby enabling the composite membrane to achieve both high-performance oil/water emulsion separation (flux exceeding 3029 L·m^−2^·h^−1^ with rejection above 99.0%) and photocatalytic self-cleaning capability (93.1–96.9% degradation efficiency of various dyes within 40 min via peroxymonosulfate activation) [164]. From a broader perspective, such multifunctional expansion represents a common developmental trajectory of metal–organic coordination network materials, with the engineering strategies developed for metal-phenolic networks offering valuable references for further functionalization of PBA-based membranes [92].

Frontier research has extended functional integration to photothermal conversion and resource recovery. The photothermal effect of MXene-containing 3D aerogels can support membrane purification by coupling solar heating with separation and photocatalytic degradation [72]. Meanwhile, the selective recovery of precious metal ions (Ag, Au, Pd) from oil/water emulsions represents a paradigm shift from pollutant elimination to resource valorization, expanding the conceptual scope of multifunctional membranes beyond mere remediation toward circular economy principles [73].

The residual oxygen-containing functional groups and intrinsic defect sites on reduced graphene oxide (RGO) sheets act as highly efficient electron transfer mediators, a “molecular bridge”, that facilitate the separation and migration of photogenerated electron-hole pairs from the Ag-TiO_2_ photocatalyst, thereby significantly enhancing its visible-light-driven photocatalytic performance and enabling the membrane to achieve a water flux of 191 L·m^−2^·h^−1^ with nearly 100% rejection [165]. Building upon this foundation, biomimetic interface engineering strategies, particularly PDA with its abundant catechol functional groups, have emerged as a universal platform for constructing highly efficient, synergistically catalytic heterointerfaces. The PDA-mediated RGO/PDA/g-C_3_N_4_ composite membrane achieved a methylene blue retention rate of 99.8% [166]; the RGO/PDA/Bi_12_O_17_Cl_2_ composite membrane exhibited broad-spectrum catalytic activity, achieving 98% removal of methylene blue within 100 min and 96% removal of 4-chlorophenol within 160 min, with stable recyclability over 10 cycles [167]; and the more complex ternary RGO/PDA/Bi_12_O_17_Cl_2_-TiO_2_ heterojunction membrane achieved complete photodegradation of both pollutants [168]. A fundamental trade-off exists between catalytic efficiency and system complexity: while the binary system achieved exceptional pollutant retention, the ternary system traded increased fabrication complexity for more thorough mineralization capability [166,168].

A distinctive synergistic mechanism emerges in systems where catalytic reactions generate gaseous byproducts that actively enhance separation performance. In a carbonaceous-silver nanofibrous membrane, gas bubbles generated from the catalytic reaction promote floatation demulsification, establishing a positive feedback coupling between catalysis and separation that further enhances overall treatment efficiency: the carbonaceous-silver nanofibrous membrane shows a permeability of 45,612 ± 430 L·m^−2^·h^−1^·bar^−1^ for toluene-in-water emulsions with separation efficiencies above 99%, while adsorbing more than 90% of Pb^2+^ and degrading nearly 100% of methylene blue via Fenton-like oxidation. This represents a particularly elegant form of synergy where the catalytic process not only degrades pollutants but also physically assists the separation function through bubble generation, creating a self-amplifying loop that transcends simple additive effects. Although this mechanism has been demonstrated primarily in carbon-based nanofibrous systems, the underlying principle, selecting gas-evolving catalysts to actively enhance demulsification, offers a translatable blueprint for the design of 3D graphene assemblies and other catalytic membrane architectures [169].

Table 3 groups the photocatalytic materials by activation mode. The comparison reveals the breadth of the photocatalytic systems: most operate under visible light, reflecting progress in bandgap engineering.

### 2.4. Performance Enhancement and Stability Improvement Strategies

The intrinsic instability of ZnO in acidic and strongly alkaline solutions poses a fundamental challenge for its application in photocatalytic membranes. Strategies to address this limitation have primarily followed two complementary design paradigms: physical encapsulation within chemically inert polymer shells and the construction of multi-component composite architectures. Ding et al. demonstrated that a ZnO/PVDF core–shell structure membrane maintained normal rhodamine B degradation activity after acid and alkali treatment, validating physical shielding as a viable stability-enhancing paradigm [170]. Lu et al. prepared a ZnO/polyaniline (PANI)/PAN nanofibrous membrane that maintained stable underwater superoleophobicity and hydrophilicity under adverse conditions [60], while Jiang et al. integrated CoS_x_ into a membrane matrix and demonstrated exceptional stability after submersion in various pH levels and seawater for one week [171]. A critical nuance emerges when comparing these strategies: the ZnO/PANI/PAN system relies on the electronic synergistic effect between ZnO and PANI for functionality [60], whereas the CoS_x_-based membrane achieves stability through a different, non-ZnO photocatalytic material system [171]. Beyond ZnO-based systems, MXene lamellar membranes have improved operational stability through nano-intercalation and interfacial engineering [107]. LDH-constructed surfaces maintain high separation efficiencies under varying pH conditions [116] and have demonstrated stable reuse over repeated cycles [124], supporting their baseline environmental durability.

The fabrication of continuous and dense MOF layers on flexible polymer substrates is a critical prerequisite for practical application, yet conventional synthesis methods often lead to non-uniform crystal growth and micro-cracks. Wu and colleagues addressed this challenge through an electrochemical synthesis method on polyethersulfone (PES) substrates, demonstrating that the synergistic optimization of voltage, stirring speed, and reaction time enables the formation of uniform HKUST-1 crystals and dense membrane layers. The optimized membrane maintained superior separation performance even after being rolled up 10 times with a curvature of 200 m^−1^, providing direct evidence of “ultra-flexibility” and establishing a replicable benchmark for evaluating mechanical durability [172].

The hydration layer formation mechanism and its role in achieving extreme wettability (superhydrophilicity with water contact angle of 0° and underwater superoleophobicity with oil contact angle > 150°) have been detailed in Section 2.2.1. From an antifouling performance perspective, this hydration layer prevents oil-droplet adhesion by retaining foulants only on the membrane surface, enabling facile regeneration through simple rinsing or photo-Fenton self-cleaning [127]. However, the practical utility of hydration layers is fundamentally constrained by the mechanical fragility of conventional hydrogel coatings. Liu et al. addressed this challenge through a double-network hydrogel strategy [128].

Photocatalytic self-cleaning surfaces offer an alternative pathway for membrane regeneration, relying on the generation of reactive oxygen species by semiconductor materials upon light irradiation to degrade organic foulants in situ [37,170]. TiO_2_ has been modified through metal doping, noble metal deposition, or hybridization to extend visible-light responsiveness [134,165,170], while emerging materials including ZnO, ZIF-8, g-C_3_N_4_, and β-FeOOH have also demonstrated effective self-cleaning performance [32,75,145,173]. Photocatalytic self-cleaning membranes maintain excellent separation efficiency and flux recovery over up to 100 operational cycles [26,32,135]. Long-term operation inevitably leads to progressive flux decline due to the continuous accumulation of foulants on the membrane surface and within the pores. These long-term data underscore the critical role of self-cleaning capability in sustaining membrane performance under continuous operation.

**Figure 7:**
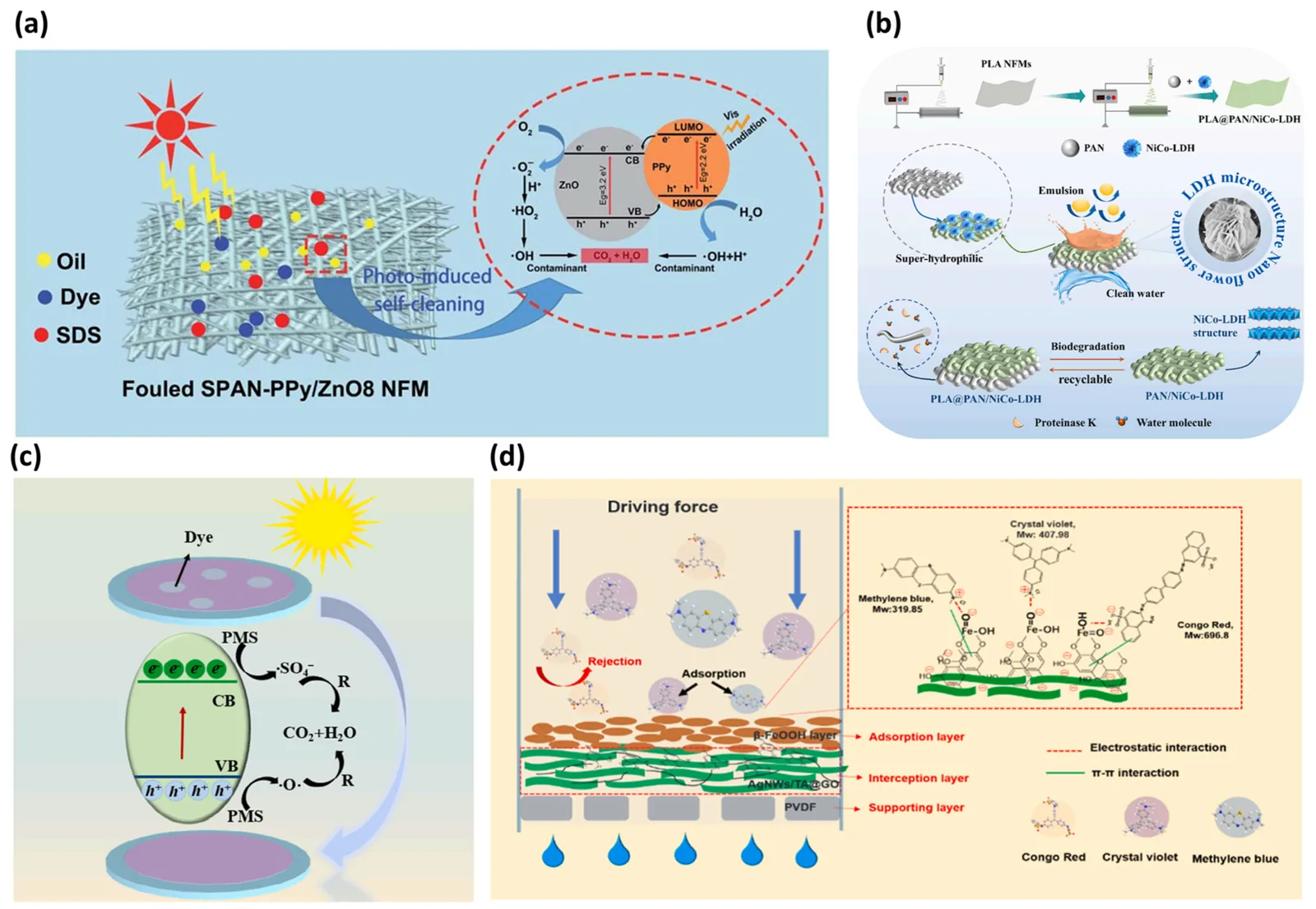
Photocatalytic self-cleaning and dye-removal mechanisms of multifunctional membranes. (**a**) Photo-induced self-cleaning of the SPAN–PPy/ZnO8 membrane; (**b**) preparation and operation of the PLA@PAN/NiCo-LDH membrane; (**c**) photocatalytic reaction pathway; (**d**) dye-removal mechanism of the multilayer β-FeOOH@GO/AgNWs-1 membrane. Reprinted from [60] for panel (**a**), [177] for panel (**b**), [171] for panel (**c**), and [32] for panel (**d**).

Four complementary approaches have emerged for flux recovery and pollution reversal. The first pathway, based on Fenton and photo-Fenton chemistry, integrates iron-based or bimetallic catalytic systems into membrane architectures. A β-FeOOH/CuFeO_2_ dual-catalyst configuration achieved an ultrahigh flux recovery ratio of 98.2% and reduced irreversible fouling ratio to 2.0% [154]. The second pathway leverages photocatalytic self-cleaning: an oxygen vacancy-engineered carbon/MnO_2−x_ nanofibrous membrane achieved 100% permeation flux recovery after 40 cycles [174]. The third pathway introduces smart responsive surfaces that enable rapid switching between superhydrophobicity and superhydrophilicity upon external stimuli, maintaining stable reversibility over more than 10 cycles [175]. The fourth pathway adopts a prophylactic design philosophy: a “dual-defense” spatially hierarchical coating achieved 99.8% emulsion separation efficiency and >90% water flux recovery after cyclic separation [176]. Figure 7 details the second of these four pathways. Three variants of catalytic self-cleaning are shown ROS generation on SPAN–PPy/ZnO (Figure 7a), photocatalysis–AOP synergy on PLA@PAN/NiCo-LDH (Figure 7b), and interlayer charge transfer in the β-FeOOH@GO/AgNWs multilayer (Figure 7d), together with the generic reaction pathway (Figure 7c).

Table 4 compares the main fouling mechanisms of representative membranes together with the corresponding antifouling or self-cleaning strategies. The table shows that fouling control can be achieved through three complementary strategies surface wettability design, oxidative degradation of foulants, and structural engineering and that effective antifouling usually integrates several of them rather than relying on a single one.

Surface engineering through modification with low-surface-energy materials enables simultaneous enhancement of interfacial stability and service durability. Deng and co-workers demonstrated that TiO_2_-SiO_2_@polydimethylsiloxane (PDMS) hybrid films retained superhydrophobic and photocatalytic properties up to 400 °C, while exhibiting resistance to strong acid attack and repeated washing [178]. Wei et al. employed a stable oxidized poly(arylene sulfide sulfone) (OPASS) polymer matrix combined with UiO-66 MOFs to achieve 99.9% regeneration efficiency over ten cycles in corrosive solutions while removing 98.44% of oil at a flux of 636 L·m^−2^·h^−1^ from both oil/water and water-in-dichloromethane emulsions [179]. Zhu et al. extended the applicable pH range of photo-Fenton membranes to 2–12 by constructing PES/Fe_3_S_4_@Al_2_O_3_ membranes [31]. Wu et al. reported that PLA@PAN/NiCo-LDH membranes maintained >99% oil rejection and 89.2% flux recovery after 30 cycles and 30 days of immersion in strong acids, bases, and high salt concentrations [177]. High salinity constitutes another critical factor in real produced water and industrial wastewater, since the abundant ions compress the electrical double layer and weaken the electrostatic repulsion between oil droplets and the membrane surface, thereby promoting fouling. These results demonstrate that LDH-based membranes are promising candidates for the treatment of high-salinity oily wastewater [114].

Industrial solid waste, fly ash, was utilized to fabricate a graphene oxide/geopolymer composite membrane achieving separation efficiencies ≥ 98.2% under gravity-driven conditions [105]. Regarding fabrication protocols, green strategies including seed-assisted in situ growth [46], hydrolytic co-deposition [46], and aqueous-phase coordination bonding [96] have been developed to eliminate toxic organic solvents. Life-cycle assessment (LCA) quantitatively demonstrated that one-step deep eutectic solvent treatment introduces fewer environmental impacts than conventional two-step methods [104]. The biodegradability of the PLA@ZIF-8 membrane provides ultimate validation of sustainability [103], demonstrating that closing the material loop, from renewable feedstock to functional membrane to harmless degradation products, is an achievable goal.

Beyond the improvements in separation performance and operational stability, certain membrane systems have already demonstrated preliminary potential for engineering scale-up. Multifunctional membranes constructed on commercial nonwoven fabrics, textiles, filter paper, and metal meshes [61,63,87,114] can be fabricated via melt-blowing, dip-coating, spray-coating, or in situ growth [46,61,63], thus exhibiting good compatibility with large-area processing techniques. 3D foams and wood-based membranes also feature gravity-driven filtration, monolithic operation, and repeated use [3,35,45,85]. In contrast, vacuum-assembled lamellar membranes [29,69] and multistep-constructed catalytic membranes [49], despite their excellent laboratory-scale performance, currently possess relatively lower technological readiness. Overall, existing studies have identified several substrates and fabrication routes with potential scale-up value, but their industrial feasibility still requires further validation [8,109].

Table 5 compares representative material systems for multifunctional membranes in terms of their advantages, limitations, flux, separation and degradation efficiencies, and stability.

Asymmetry concerns the stability column. ‘A cycle’ is not defined consistently: for the gravity-driven foams it denotes a separation run, for the photocatalytic membranes a separation-plus-irradiation sequence, and for TA@CoFe-LDH/PVDF a filtration run under applied pressure. A membrane that survives 35 cycles of gravity-driven filtration and one that survives 10 cycles of pressurized cross-flow operation have not been subjected to comparable stress. Until cycle definitions and the accompanying hydraulic conditions are standardized, cycle count should be treated as a qualitative indicator of durability rather than as a comparable metric.

## 3. Summary and Outlook

### 3.1. Full Text Summary

This paper systematically reviews the research progress of multifunctional membrane materials that integrate oil/water separation and catalytic degradation of organic pollutants, providing a comprehensive overview from design strategies, mechanisms of action, performance optimization to application prospects. The study shows that through material structure design, interface wettability regulation, and precise integration of catalytic functions, multifunctional membranes have successfully overcome the technical bottlenecks of traditional separation membranes, which are functionally single and prone to fouling. They have achieved a synergistic unification of “physical separation—catalytic degradation—membrane self-cleaning,” providing a new solution for the efficient and intensive treatment of complex oily organic wastewater.

In terms of material construction, researchers have developed multifunctional membrane materials with multiple dimensions and systems: metal oxide-based membranes are the most mature, with their visible light response and catalytic performance continuously improved through nanostructure regulation, element doping, and heterostructure construction; 2D material-based membranes have shown great potential in flux enhancement and functional integration due to their unique layered channels and large specific surface area advantages; 3D structure-based membranes further enhance mass transfer efficiency and the exposure of catalytic sites by constructing interconnected porous networks; biomass-based and green modified membranes provide new directions for the development of low-cost, environmentally friendly membrane materials.

In terms of mechanisms of action, the oil/water separation mechanism has evolved from a single wettability selectivity to a synergistic mechanism of “size sieving-emulsification coalescence-hydration layer antifouling.” The catalytic degradation mechanism covers multiple pathways such as photocatalysis, Fenton-like catalysis, and persulfate activation. Moreover, strategies such as heterojunctions and band engineering have effectively improved charge separation efficiency and catalytic activity. The synergistic coupling of adsorption and catalysis further enhances the enrichment and degradation process of pollutants, improving the overall treatment efficiency.

In terms of performance optimization, through strategies such as multi-level pore structure regulation, superwetting interface design, and low-adhesion surface construction, the separation flux and antifouling performance of the membrane have been significantly improved. Through material composites and structural optimization, the chemical stability, mechanical strength, and cycle life of the membrane have also been effectively enhanced, laying a foundation for practical applications.

### 3.2. Existing Challenges

Although the research on multifunctional separation membranes has achieved fruitful results, there are still many urgent problems and challenges to be solved when moving from laboratory-scale studies to large-scale industrial applications:

First, there are bottlenecks in large-scale preparation technology. This gap is already visible in Table 1, where technological maturity is the single column in which conventional membranes uniformly outperform multifunctional system reviewed. Currently, the multifunctional membranes prepared in the laboratory are mostly small-sized and batch samples. The preparation processes are often complex and require harsh conditions, and some functional materials are costly, making low-cost large-scale production difficult. At the same time, during the scale-up preparation process, the uniformity of the functional coating and the adhesion between the coating and the substrate are difficult to stably ensure, leading to issues such as coating peeling and performance inconsistency, which cannot meet the requirements for the preparation of industrial membrane modules [8,109].

Second, insufficient adaptability to actual complex wastewater. Most existing studies focus on simulated wastewater prepared with a single oil type and a single pollutant, under relatively ideal experimental conditions. However, actual industrial wastewater often contains multiple coexisting pollutants, high salinity, wide pH fluctuations, and suspended solids, which can significantly affect the membrane’s wettability, catalytic activity, and structural stability, even leading to a substantial decline in membrane performance. At the same time, the pollution evolution patterns and activity decay mechanisms of membranes during long-term operation in actual wastewater remain unclear, and the long-term operational stability needs to be verified [8,11,89].

Third, the challenge of balancing treatment efficiency and operational costs. Photocatalytic multifunctional membranes are limited by the visible light utilization rate and the recombination rate of photogenerated charge carriers of semiconductor materials, resulting in low quantum efficiency. When treating high-concentration organic wastewater, the degradation rate is slow and the treatment effect is limited, and additional light source systems are required, increasing energy consumption and costs. Fenton-like catalytic membranes require continuous addition of oxidants such as hydrogen peroxide and persulfate, leading to higher operational reagent costs. Because these systems incorporate metal-containing catalysts, including MOF-on-MOF, Co(OH)_2_, and ZIF-67 platforms, metal ion leaching and secondary-pollution risks should be explicitly measured during durability assessment rather than assumed to be negligible [22,180,181].

Fourth, the lack of a standardized evaluation system. Currently, there are significant differences in the test conditions across different studies, including operational pressure, pollutant concentration, effective membrane area, and light source parameters, with no unified standards. Additionally, the definitions and testing methods for performance evaluation indicators vary, making it difficult to conduct lateral comparisons and objective assessments of different membrane materials, which is not conducive to technological iteration and industry-standard development [47,109].

**Figure 8:**
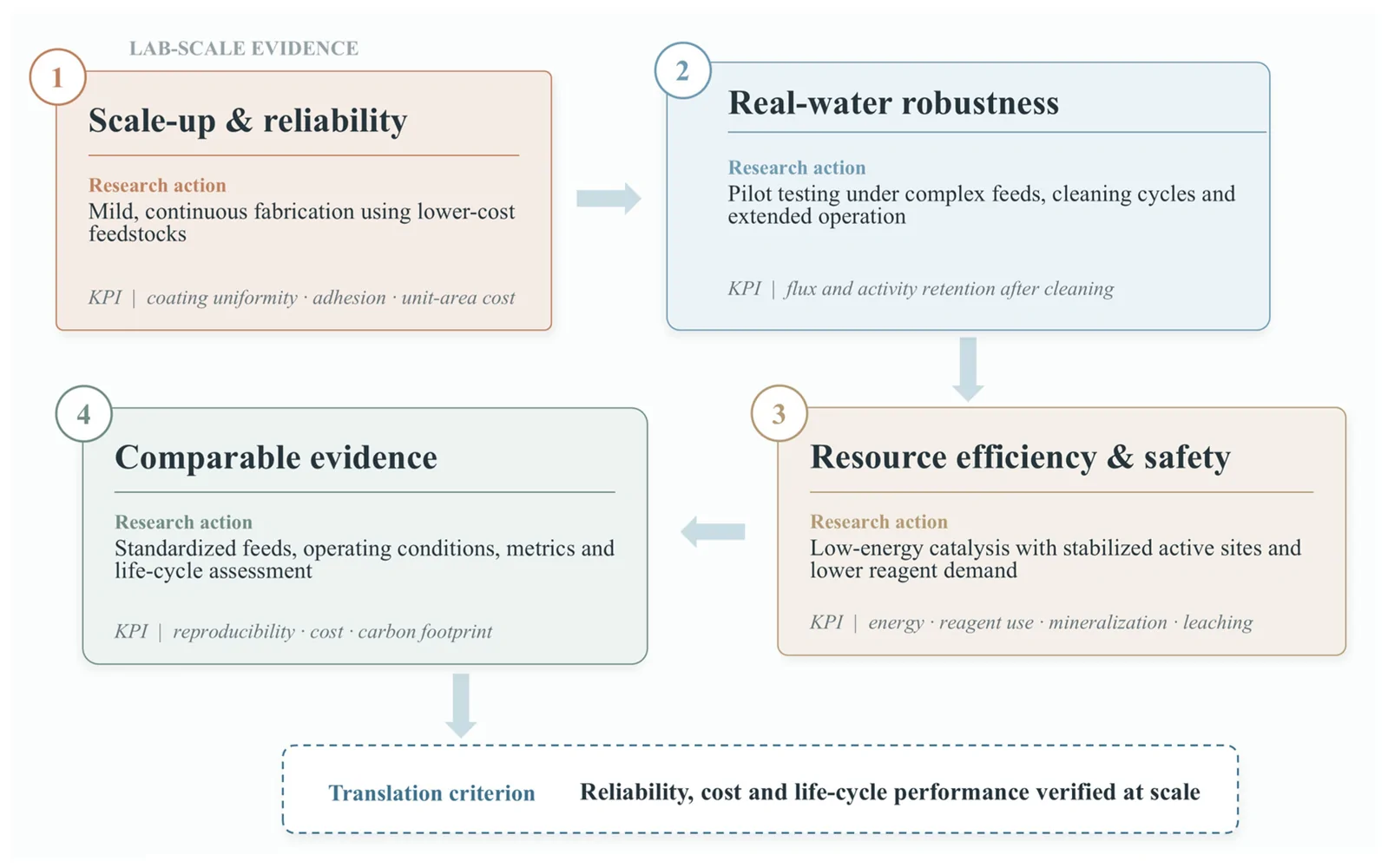
Roadmap for industrial translation of multifunctional membrane technology. The roadmap traces progress from laboratory-scale evidence and comparative testing to real-water validation, resource efficiency, safety assessment, and verification of reliability, cost, and life-cycle performance at scale.

Figure 8 maps the four challenges of Section 3.2 onto sequential validation stages. This roadmap identifies current technological readiness gaps and provides clear guidance for future research directions, including green fabrication, long-term stability, and full life-cycle assessment.

### 3.3. Future Prospects

To address the aforementioned challenges, future research and application of multifunctional separation membranes can focus on the following directions:

#### 3.3.1. Development of Green, Low-Cost, and Scalable Preparation Technologies

Further simplify membrane preparation processes and develop simple, mild, and scalable preparation methods such as dip-coating, spraying, and in situ growth to replace complex multistep synthesis processes. Actively expand the application of low-cost raw materials such as biomass and industrial solid waste-based materials, reduce the use of precious metals and toxic reagents, and lower the preparation cost of membrane materials. At the same time, explore continuous membrane preparation production lines, optimize the deposition process of functional coatings, enhance the adhesion and uniformity of coatings with substrates, ensure the performance stability of large-sized membrane modules, and lay the foundation for industrial applications [4,8,182].

#### 3.3.2. Improvement in Adaptability to Complex Water Quality and Long-Term Operational Stability

In response to the complex characteristics of actual industrial wastewater, develop broad-spectrum multifunctional membrane materials with multiple components and coupled mechanisms. Through component synergy and structural optimization, enhance the membrane’s treatment performance and structural stability in high-salinity, wide pH, and multi-pollutant coexistence systems. At the same time, strengthen pilot-scale studies on actual wastewater treatment, systematically investigate the flux decline patterns, pollution evolution characteristics, and catalytic activity retention of membranes during long-term operation, optimize membrane cleaning and regeneration processes, and establish a full-life-cycle operation and maintenance plan to ensure the reliability and economic viability of actual operations [8,11,89].

#### 3.3.3. Construction of Efficient, Low-Energy Consumption Catalytic Systems and Coupled Processes

On one hand, continuously optimize the band structure and active site design of catalytic materials. By constructing Z-type heterojunctions, introducing single-atom catalysis, and combining piezoelectric catalysis, enhance the quantum efficiency and visible light utilization of photocatalytic materials, reduce the dependence on artificial ultraviolet light sources, and make full use of natural light for energy-saving operations. On the other hand, develop high-activity and high-stability heterogeneous Fenton-like catalyst immobilization technologies to reduce metal ion leaching. At the same time, explore coupled processes of membrane separation with photoelectrochemical catalysis and biological treatment, constructing a deep treatment system of “separation-enrichment-degradation-mineralization” to improve the effluent water quality while reducing the overall operational energy consumption and costs [54,150,171].

#### 3.3.4. Development of Intelligent Multifunctional Membranes and Systems

Combine stimulus-responsive materials to develop intelligent multifunctional membranes with pH response, light response, and electric response, enabling dynamic regulation of separation performance and catalytic activity. These membranes can flexibly switch operating modes according to influent water quality and treatment requirements, enhancing adaptability to water quality fluctuations. At the same time, future systems should introduce online membrane-fouling monitoring, intelligent cleaning, and automatic optimization of operating parameters to reduce manual maintenance and improve operational stability.

#### 3.3.5. Standardized Evaluation Systems and Full-Life-Cycle Assessments

Establish a unified performance testing standard and evaluation index system for multifunctional membranes, standardize the test conditions and calculation methods for key parameters such as separation efficiency, permeation flux, degradation rate, antifouling properties, and cycle stability, providing an objective basis for lateral comparisons of different research results and technological iterations. Full-life-cycle assessment should compare low-impact preparation routes [104] with catalytic meshes that deliver recyclability but still contain metals [183], quantifying environmental and economic trade-offs from raw-material acquisition through fabrication, operation, regeneration, and end-of-life disposal. Such analysis is essential for identifying genuinely sustainable and economically feasible multifunctional membrane technologies.

In summary, multifunctional separation membranes, with their integrated and efficient technological advantages, have broad application prospects in the treatment of complex oily organic wastewater. With the continuous development of materials science, interfacial chemistry, catalytic engineering, and membrane preparation technologies, the aforementioned challenges will be gradually resolved. Multifunctional membrane technologies will surely play an increasingly important role in water environment management and water resource recycling, providing strong technical support for green and low-carbon sustainable development.

## Figures and Tables

**Table 1:** Comparison of key performance parameters between multifunctional membranes and conventional membrane technologies.

Membrane Category	Material/Representative System	Separation Efficiency/Rejection (%)	Flux (L·m^−2^·h^−1^)	Removal of Dissolved Pollutants	Antifouling/Self-Cleaning	Operating Driving Force	Technological Maturity	Refs.
Conventional ultrafiltration membrane	Commercial PVDF membrane	44.5	4008	Essentially ineffective	Irreversible fouling; non-regenerable	Pressure-driven (0.2 MPa)	Mature industrial application	[40,41]
Conventional ceramic membrane	Al_2_O_3_ microfiltration membrane	98.5	760	Essentially ineffective	Severe fouling; requires chemical cleaning	Pressure-driven (0.07–0.2 MPa)	Mature industrial application	[39]
Conventionally modified membrane	NS-PVA-PVDF	>94	5437	98%	>90% (flux recovery) after cleaning with 3 wt.% NaOH	Pressure-driven (0.08 MPa)	Laboratory stage	[40,41,42]
Multifunctional photocatalytic membrane	TiO_2_ nanofibrous membrane	>99	40,163	~96% (RhB, 1 h)	Photocatalytic self-cleaning; 98.5% after 10 cycles	Gravity/low-pressure driven	Laboratory stage	[43,44,45]
Multifunctional 3D framework	TPU/TiO_2_/PDA foam	99.6	21,035 (water)	96% (RhB, 1 h)	Photocatalytic self-cleaning; 98.5% after 10 cycles	Gravity-driven	Laboratory stage	[45]
Multifunctional biodegradable membrane	PLA@ZIF-8	>99.8	6280	92.8% (diesel) 99.7% (MB)	Photocatalytic self-cleaning; 120-day degradation in soil	Pressure-driven	Laboratory stage	[46]

**Table 2:** Summary of representative membrane materials, substrates, preparation methods, separation modes, driving forces, and treatment targets.

Membrane Material	Substrate	Preparation Method	Separation Mode	Driving Force	Treatment Target	Refs.
TiO_2_@PVDF/PAN	PVDF/PAN	Coaxial electrospinning	Dead-end filtration	Gravity/low pressure	Oil/water emulsion	[44]
GO@CoS_x_	GO	Vacuum filtration + in situ growth	Cross-flow/dead-end	Pressure-driven	MB dye + oil emulsion	[69]
Ag@MXene/PEN	PEN	Electrospinning + vacuum filtration	Dead-end filtration	Pressure-driven	Dyes + oil/water emulsion	[107]
Janus wood membrane	Natural wood	Delignification + in situ polymerization	Dead-end filtration	Gravity/capillary force	Oil/water + dyes + seawater	[35]

**Table 3:** Comparison of representative photocatalytic materials for pollutant degradation in multifunctional membranes.

Material System	Representative Photocatalytic Material	Light Response Range	Photocatalytic Conditions	Degradation Target	Degradation Efficiency	Refs.
Metal oxide	TiO_2_ nanofibers	UV light	UV irradiation	Rhodamine B	96% (1 h)	[45]
2D (GO)	RGO-Ag-TiO_2_	Visible light	Visible-light driven; RGO acts as an electron-transferring “molecular bridge”	Methylene blue	~98%	[165]
2D (MXene)	Ag@MXene/PEN fibrous membrane	Visible light	Visible light irradiation; Ag@MXene heterojunction	Methyl orange, crystal violet	95.24% (MO, 60 min) 95.61% (CV)	[107]
2D (g-C_3_N_4_)	Seaweed-like g-C_3_N_4_ self-supporting membrane	Visible light	Visible-light driven; narrow bandgap	Dyes; antibacterial	~100%	[75]
3D porous framework	TPU/TiO_2_/PDA foam	UV light	UV irradiation	Rhodamine B	96% (1 h)	[45]
3D porous framework	aPAN/MXene@Ag–Ag_2_S aerogel	Visible light	Visible light; Ag–Ag_2_S heterostructure enhances light absorption	High-concentration dyes	Efficient degradation	[72]
Biomass-based	Janus wood membrane (PPy@Ag/AgCl@wood)	Simulated sunlight	Driven by simulated sunlight	Methylene blue, Rhodamine B	86.4% (MB) 91.9% (RhB)	[35]

**Table 4:** Comparison of membrane fouling mechanisms and antifouling/self-cleaning strategies for representative membranes.

Material Class	Representative Membrane	Foulant(s)/Condition	Fouling Mechanism	Resistance/Regeneration Mechanism	Refs.
Metal oxide	β-FeOOH/CuFeO_2_-PVDF (MFeOOH/CF)	Crude oil, BSA, and methylene blue	Oil passes an unstable underwater superoleophobic layer, then adsorbs and spreads on oleophilic PVDF and pore walls.	Stable underwater superoleophobicity limits attachment; solar-driven photo-Fenton oxidation degrades residual foulants.	[154]
2D materials	TiO_2_/Co_3_O_4_/GO-coated TiO_2_ stainless steel mesh	Oil droplets in O/W emulsions	Oil-droplet adhesion at the underwater oil–water–solid interface.	Superamphiphilicity in air and underwater superoleophobicity reduce oil adhesion; the heterojunction photodegrades Congo red.	[18]
2D materials	Conventional 2D GO membrane (control)	N/A (structural control)	Interlayer hydrogen bonding causes dense GO restacking, reducing porosity and permeation flux.	No active resistance mechanism; benchmark for 3D structural regulation.	[164]
3D porous	FeCo-PBA@GO/PAN fibrous membrane	O/W emulsion and dissolved dyes	Hydrophobic PAN favors oil adhesion; dense 2D GO stacking constricts water pathways.	3D microspheres suppress GO restacking and impart underwater superoleophobicity; PMS-assisted photocatalysis degrades dyes.	[164]

**Table 5:** Comparison of representative material systems for multifunctional membranes: advantages, limitations, and key performance indicators.

Material System	Representative Material	Advantages	Limitations	Flux (L·m^−2^·h^−1^)	Separation Efficiency (%)	Degradation Efficiency (%)	Stability/Cycling Number	Refs.
Metal oxide	TPU/TiO_2_/PDA 3D porous foam	Gravity-driven high flux; micro/nano pores formed by particle accumulation	UV-driven; visible-light performance not clarified	21,035 (water) 14,500 (oil)	99.6	96 (RhB, 1 h, UV)	Separation efficiency remained at 98.5% after 10 cycles	[45]
2D (GO)	TiO_2_ nanowires/GO	Nanowire intercalation; uniform interlayer distribution	Long-term stability challenges	273	>98 (dye rejection)	—	5 cycles with stable flux and >98% removal	[129]
2D (MXene)	Ag@MXene/PEN fibrous membrane	Metallic conductivity; photothermal/antibacterial; heterojunction synergy	Challenges in functional group controllability	—	>99.4	95.24% (MO) 95.61% (CV) ~99.99% antibacterial (*E. coli*)	Enhanced long-term operational stability	[107]
2D (g-C_3_N_4_)	Seaweed-like g-C_3_N_4_ self-supporting membrane	Substrate-free/binder-free; high roughness; large specific surface area	Low quantum efficiency; rapid recombination	3114.0 (L·m^−2^·h^−1^·bar^−1^)	97.4	~100% (dyes) ~100% (antibacterial)	Stable over 35 cycles	[75]
2D (MOF)	UiO-66-NH_2_@h-PPS	Assembly of hydrophilic MOF nanosheets; micro/nano hierarchical structure	—	7638.1	99.1	85.7 (MB, 120 min, visible light)	Stable over 20 cycles	[79]
3D porous framework	Calcium alginate gel beads	Interconnected macropores; efficient mass transfer; “nanoreactor”	Multi-component pre-gelation; two-step crosslinking	—	—	~99% (RhB, 30 min, visible light)	—	[83]
Biomass-based	TiO_2_/cotton fabric	Natural cellulose; green and renewable	—	6800	>99.90	98.0 (MB, visible light)	20 cycles with >99.8%	[87]
MOF-based	NH_2_-MIL-125/PAA	Synergistic engineering of ligands and metal clusters	—	—	>99	>99	>99% removal maintained over 10 cycles	[149]
LDH-based	TA@CoFe-LDH/PVDF	PMS activation; dynamic filtration accelerates kinetics	TA blocks membrane pores; dependence on TA; slow in static mode; high pressure drop; metal leaching	529.8 (dead-end); 593.9 (cross-flow)	99.0% (91–95% without TA)	~100% (MB, 5 min, PMS)	Degradation rate under dynamic filtration was 23.89× that under static conditions	[161]
Fenton-like	Carbon/MnO_2−X_ nanofibers	Oxygen vacancy engineering	—	Up to 41,557 (surfactant-free O/W)	≥99.3	Complete degradation of MB/RhB within 20 min under visible light	100% flux recovery after 40 cycles	[174]

## Data Availability

No new data were created or analyzed in this study. Data sharing is not applicable to this article.
